# Just-in-Time Feedback in Diet and Physical Activity Interventions: Systematic Review and Practical Design Framework

**DOI:** 10.2196/jmir.8701

**Published:** 2018-03-22

**Authors:** Susan M Schembre, Yue Liao, Michael C Robertson, Genevieve Fridlund Dunton, Jacqueline Kerr, Meghan E Haffey, Taylor Burnett, Karen Basen-Engquist, Rachel S Hicklen

**Affiliations:** ^1^ Department of Behavioral Science Division of Cancer Control and Population Sciences The University of Texas MD Anderson Cancer Center Houston, TX United States; ^2^ Institute for Health Promotion & Disease Prevention Department of Preventive Medicine University of Southern California Los Angeles, CA United States; ^3^ Division of Behavioral Medicine Department of Family Medicine and Public Health University of California, San Diego San Diego, CA United States; ^4^ Department of Epidemiology University of Texas School of Public Health The University of Texas Health Science Center at Houston Houston, TX United States; ^5^ Department of Family and Consumer Sciences College of Health Science Sam Houston State University Huntsville, TX United States; ^6^ Research Medical Library The University of Texas MD Anderson Cancer Center Houston, TX United States

**Keywords:** health behavior, diet, exercise, task performance and analysis, Internet, mHealth, accelerometer, activity monitor, self-tracking, wearable sensors

## Abstract

**Background:**

The integration of body-worn sensors with mobile devices presents a tremendous opportunity to improve just-in-time behavioral interventions by enhancing bidirectional communication between investigators and their participants. This approach can be used to deliver supportive feedback at critical moments to optimize the attainment of health behavior goals.

**Objective:**

The goals of this systematic review were to summarize data on the content characteristics of feedback messaging used in diet and physical activity (PA) interventions and to develop a practical framework for designing just-in-time feedback for behavioral interventions.

**Methods:**

Interventions that included just-in-time feedback on PA, sedentary behavior, or dietary intake were eligible for inclusion. Feedback content and efficacy data were synthesized descriptively.

**Results:**

The review included 31 studies (15/31, 48%, targeting PA or sedentary behavior only; 13/31, 42%, targeting diet and PA; and 3/31, 10%, targeting diet only). All studies used just-in-time feedback, 30 (97%, 30/31) used personalized feedback, and 24 (78%, 24/31) used goal-oriented feedback, but only 5 (16%, 5/31) used actionable feedback. Of the 9 studies that tested the efficacy of providing feedback to promote behavior change, 4 reported significant improvements in health behavior. In 3 of these 4 studies, feedback was continuously available, goal-oriented, or actionable.

**Conclusions:**

Feedback that was continuously available, personalized, and actionable relative to a known behavioral objective was prominent in intervention studies with significant behavior change outcomes. Future research should determine whether all or some of these characteristics are needed to optimize the effect of feedback in just-in-time interventions.

## Introduction

Recent advancements in technology, particularly the advent of activity monitors and other wearable body sensors, have the potential to influence innovations in diet and physical activity (PA) assessment and interventions. According to 2013 Pew statistics [1], 7 in 10 US adults report tracking at least one health indicator (eg, weight, diet, exercise, blood pressure, blood sugar, or sleep patterns), and of people who track a health indicator, 46% report that such tracking has changed their approach to health maintenance, 40% say it has led them to engage with health care providers, and 34% say it has affected a decision on how to treat an illness or condition. However, only 21% of people who track a health indicator say that they used some form of technology to assist their tracking efforts. These data highlight that health-tracking technology is surprisingly underutilized as a resource to motivate health behavior change. However, studies testing the efficacy of health-tracking technology to motivate behavior change alone or as part of a theory-based behavioral intervention have shown that such technology-based approaches produce null to modest short-term improvements in health behaviors or weight loss compared with traditional approaches (eg, in-person coaching and telephone coaching) [2-4]. One potential reason for these lackluster findings is that our current behavioral theories have not yet been adapted to leverage the advantages of health-tracking technologies.

Wearable sensors, particularly Internet-connected sensors, can dramatically enrich the temporality and frequency of health behavior data collection by facilitating self-monitoring and reducing self-report biases. Another important but less realized advantage of wearable sensor technology is its bidirectional communication capability. The latest health trackers are equipped with interactive software apps housing algorithms that allow data to be processed in real time to deliver actionable feedback at critical moments in a person’s daily life to facilitate the attainment of predetermined health behavior goals. These features are likely to enhance bidirectional communication between investigators and their study participants or between patients and their health care providers, thereby improving the users’ engagement with the technology and subsequently facilitating intervention adherence and improving health outcomes. Technology-enhanced interventions are likely the future of behavior change research; however, the use of fast-advancing technologies that enable just-in-time interventions is outpacing the adaptation of theory-based intervention design [5].

Performance feedback is a key, theory-based behavior change strategy [5] that has not been optimally adapted for technology-enhanced interventions. Performance feedback is historically defined as actions taken by (an) external agent(s) to provide information regarding some aspect(s) of one’s task-specific performance [6]. Several behavior change theories, including control theory [7,8], goal-setting theory [9], and social cognitive theory (SCT) [10], deem feedback to be an important component of successful behavior change. Although the rationale for using feedback varies across these theories, each characterizes feedback as a self-regulation strategy that reveals to people their progress in relation to their goal(s) [7-10]. In addition, the feedback intervention theory (FIT) posits that feedback, as a component of behavioral interventions, motivates behavior change by focusing one’s attention on the behavioral task itself [6], which introduces the importance of timing in the delivery of feedback messages. Collectively, these theories indicate that feedback should be personalized and goal-oriented and presented when attention could be refocused to improve the likelihood of goal attainment. These characteristics are consistent with the strategy of using feedback in just-in-time behavior change interventions [5,11,12]. Just-in-time interventions are those which are delivered when there is an opportunity for positive change. Performance feedback represents a type of support that can be delivered at important decision points relative to a specific behavior and the attainment of related behavioral goals. However, the use of feedback and its content varies widely within behavioral domains, such as learning, professional care practice, and employee performance [13]. Furthermore, there is limited systematic analysis of the characteristics and use of feedback in health behavior interventions. The paucity of literature in the area of health behavior change might be limiting our ability to optimize feedback content to obtain the greatest intervention effect.

Several studies have reviewed the efficacy of digital health technology to promote weight control, PA, and healthy diets. These reviews generally support the use of technology for self-monitoring and intervention delivery, but they also acknowledge that the content and design of future interventions will need more rigorous evaluation to optimize their effects [12,14-16]. In a historical perspective and meta-analysis, Kluger and Denisi [6] found that feedback interventions significantly improved performance. The authors concluded that feedback that is more likely to have a positive effect on behavior change is specific to a familiar task (ie, personalized) and attracts attention to discrepancies between current performance and a desired goal or target at the task level (ie, goal-oriented). They further suggested that feedback should contain cues that support one’s performance of the task (ie, should be action-oriented). In a more recent systematic review and meta-analysis, Sherrington and colleagues [17] showed that participants enrolled in weight loss interventions that provided personalized feedback lost on average 2.13 kg (*P*<.001) more weight than those in control groups who received no feedback. Although their review provided evidence that feedback was an effective behavior change strategy in weight loss interventions, it lacked an in-depth characterization of effective feedback components to be applied in future studies. Collectively, these reviews support the use of feedback to motivate health behavior change, particularly in the context of diet and PA, and point to key characteristics of potentially effective feedback. To date, however, there has been no framework developed for designing feedback to be used technology-enhanced behavioral interventions.

The primary goal of this review was to (1) Provide a review of diet and physical activity (PA) interventions that use just-in-time feedback as a behavior change technique (BCT); (2) Characterize key aspects of the reviewed studies’ feedback content characteristics, prompting style, and delivery methods; and (3) describe how the implementation of these key aspects differed by studies that found significant effects of using feedback to motivate behavior change (intervention efficacy). Our secondary goal was to develop a practical framework for designing feedback that could be incorporated into technology-enhanced just-in-time interventions. We focused on feedback characteristics inferred from the theoretical and historical foundations of the use of performance feedback as a health behavior change strategy: timeliness, personalization, action orientation, and goal orientation.

## Methods

### Literature Search

Two authors (SMS and YL) with the assistance of a medical librarian (RSH) devised systematic strategies to search the Ovid MEDLINE, Ovid EMBASE, PubMed, Cochrane Library, Scopus, and PsychInfo databases for all relevant literature published through December 2016. Searches were limited articles written in the English language and conducted in humans. Database search strategies included the use of controlled vocabulary (eg, Medical Subject Headings and Emtree) and keywords to identify studies addressing PA or diet in conjunction with feedback. Keywords included physical activity, exercise, diet, eating, intervention, and feedback. Additionally, the bibliographies of topically relevant review papers and all included studies were examined to identify any additional studies.

### Study Inclusion and Exclusion Criteria

Eligible studies included (1) Just-in-time feedback as an intervention component and (2) Targeted behavior changes that included PA, sedentary behavior, or dietary intake. Just-in-time feedback was defined on a case-by-case basis as any feedback that focused on participants’ daily PA, sedentary behavior, or dietary intake and that was provided within 1 min to 1 day of assessing current performance, as appropriate to each intervention or behavior change goal. Studies with multiple feedback components were included, but at least one type of delivered feedback must have met this definition of just-in-time feedback.

Studies were excluded if (1) the intervention-targeted behaviors were off-topic (eg, studies of clinical education, personnel, management, medication adherence, blood glucose self-monitoring, and symptom management); (2) no intervention outcome results were reported (eg, protocol papers); (3) the time frame for providing feedback was greater than 1 day (eg, weekly performance summaries); (4) no or inadequate feedback was provided (eg, studies of performance tracking without evaluation); or (5) they reported the secondary outcomes of an included intervention.

### Data Extraction and Management

Data were extracted into a structured coding form according to Preferred Reporting of Systematic Reviews and Meta-Analyses (PRISMA) guidelines [18] and the Cochrane Handbook for Systematic Reviews of Interventions [19]. Five reviewers (SMS, YL, MCR, MEH, and TB) extracted characteristics and outcomes from all studies using a standardized data extraction form. The following information was extracted: (1) general study characteristics (ie, country of study, study type, participant population, participant demographics, and study sample size); (2) intervention characteristics (ie, intervention name, study design, intervention duration, behavioral theories used, and intervention goals); (3) just-in-time feedback characteristics (eg, content, delivery frequency, and delivery mode); and (4) intervention results (ie, within-group changes and between-group comparisons in targeted behaviors and weight outcomes). A copy of the data extraction form is provided as Multimedia Appendix 1. A comparison of the extracted data across reviewers was conducted (SMS and YL). Differences in the extracted data were resolved by a discussion between the expert reviewers (SMS and YL) to complete the dataset.

Assessing each study for risk of bias was performed using the 2010 version of the Quality Assessment Tool for Quantitative Studies [20]. An overall quality score for each study was assigned based on the ratings for six domains: (1) selection bias, (2) study design, (3) confounders, (4) blinding, (5) data collection methods, and (6) withdrawals and dropouts. The information extraction and quality assessment for each study was performed independently by two reviewers (YL and MCR). Discrepancies between reviewers’ ratings were resolved through discussions that led to a consensus (YL, MCR, and SMS).

### Analysis

A meta-analysis was not possible owing to substantial heterogeneity in study design, study quality, intervention type, and outcome measures, as well as a lack of studies that explicitly tested the efficacy of using feedback as a BCT. Furthermore, the primary and secondary outcomes of the studies varied widely. We limited our summary of results to primary and secondary study outcomes that were specific to changes in PA, dietary intake, or body weight or body composition. Consistent with the design of a previous review [6], two key criteria were used to determine whether a study explicitly tested feedback: (1) the study had at least one treatment group that received feedback that was not confounded with other manipulations (not matched in the control group) and (2) the study included at least one control group or quasi-control group that received no feedback. Data were synthesized narratively rather than quantitatively.

## Results

### Literature Search

**Figure 1:**
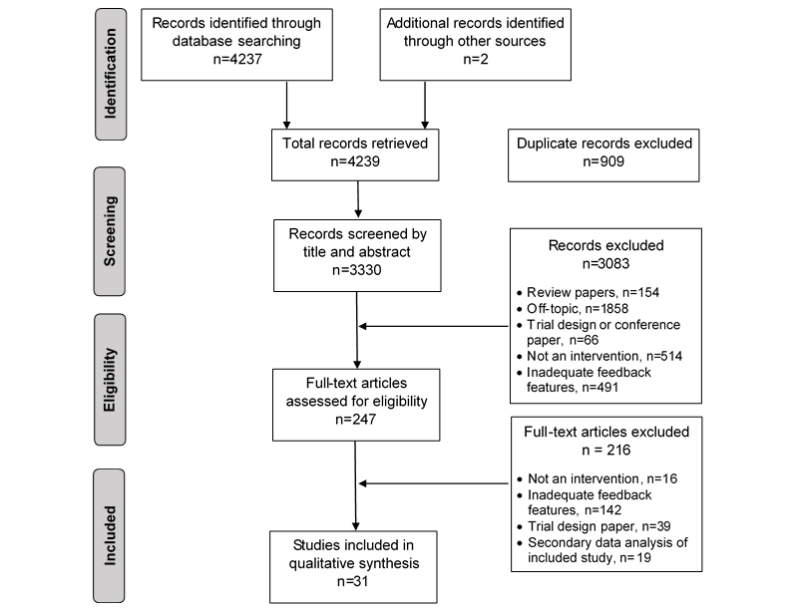
Preferred Reporting of Systematic Reviews and Meta-Analyses (PRISMA) diagram.

The literature search yielded 4239 studies, of which 909 were duplicates, leaving 3330 articles to be screened for eligibility. A total of 3083 articles were excluded upon title or abstract screening because they were unrelated to diet or PA, had no reported outcomes, were nonintervention studies, or had ineligible feedback features. Thus, 246 full-text articles were assessed for eligibility. After 215 articles that did not meet the inclusion criteria were excluded, 31 studies with a total of 6623 participants were included in the review (see PRISMA diagram, Figure 1).

### Characteristics of Included Studies

The studies’ characteristics are summarized in Multimedia Appendix 2. Studies varied by the behavior about which feedback was provided and by their sample size, population, design, and duration. Of the 31 studies, 3 focused on diet- or nutrition-related behavior only [21-23], 15 focused on PA or sedentary behavior only [24-38], and 13 focused on both diet and PA [39-51]. The median number of study participants was 83 (range=10-1488). Studies were conducted in the United States, the United Kingdom, the Netherlands, Australia, Belgium, Denmark, Hong Kong, Ireland, Japan, Portugal, and South Korea and included adults (≥18 years), children (<18 years), or young adults (17-26 years). The participants’ weight statuses were not consistently reported, but at least 11 studies enrolled only overweight or obese individuals [21,22,24,25,39,40,43,47,49-51]. A total of 28 studies were randomized controlled trials (level of evidence I) [21-24,26,27,29-41,43-50], 5 of which used clustered randomization [23,31,32,35,36] (level of evidence I). The 4 remaining studies used within-subjects single-arm [28,42,51] or counterbalanced designs [25] (level of evidence II). Study duration ranged from 2 weeks to 24 months. Four studies [31,32,35,41] had postintervention follow-up periods that ranged from 4 weeks to 6 months.

### Risk of Bias

The 31 studies’ risks of bias are summarized in Multimedia Appendix 3. Using the current Quality Assessment Tool for Quantitative Studies [20], we determined that 18 studies had a moderate global rating, 9 had a weak global rating, and 4 had a strong global rating. All but 4 studies received a strong study design rating for being randomized controlled trials. A total of 25 studies received strong scores for controlling for potentially confounding variables, 23 studies used data collection measures with demonstrated reliability and validity, and 19 studies had retention rates of ≥80% across conditions. The risk of selection bias posed the greatest threat to validity; 22 studies received a weak score in this domain. Blinding was rated as weak in 4 studies; however, the assessment tool we used may have underestimated this bias [52]. Most studies did not describe blinding procedures for research staff or participants.

### Feedback Features of Included Studies

The 31 studies varied in the content, frequency, timing, and delivery of feedback, as well as in the methods used to monitor targeted behaviors and the theoretical foundations that guided the feedback content. Multimedia Appendix 4 provides a full description of the feedback features the studies used and a summary of the key features described below.

#### Theoretical Foundation

In 24 studies [22-24,26,27,29-31,33-44,46,48-50], behavior change theories or guiding principles were used as the foundation for the use of feedback as a BCT. The most frequently endorsed behavior change theories was the SCT (9 studies) [23,27,29,31,39-41,48,49]. Other theories that were endorsed by at least two studies included the control theory [33,34], the health belief model [31], and the transtheoretical model or Stages of Change [38,49]. A total of 8 studies [22,30,31,35-37,42,43] reported using a combination of three or more theories or guiding principles. A total of 7 studies [21,25,28,32,45,47,51] did not specify a guiding theory.

#### Feedback Content

By its design, this review included only studies that delivered just-in-time feedback. In 10 studies [21,25,30,31, 33,35-37,43,49], feedback was available continuously, in 3 [27,45,46], feedback was provided at multiple times daily, and in 18 [22-24,26,28,29,32,34,38-42,44,47,48,50,51,53], feedback was provided once daily. All but one study [32], which provided team-based feedback, gave *personalized* feedback that reflected the person’s own performance. Feedback also often included performance summary information; however, not all feedback was *goal-oriented*. A total of 24 studies [21-25,27-30, 33-35,37-41,44-48,50,51] provided graphical or other visual or verbal feedback on performance relative to known goals or targets, and 8 studies [26,31,32,36,42,43,49] incorporated only raw performance summaries into the feedback. Of the 24 studies that provided goal-oriented feedback, 12 [21-25,27-29, 33,38,45,46] had either self-selected, incremental, or adaptive goals, and 12 [30,34,35,37,39-41,44,47,48,50,51] utilized static goals, targets, or published recommendations (eg, 10,000 steps per day). Goal-oriented feedback that referenced standardized or adaptive thresholds or recommendations was either cumulative (eg, progress toward daily goals) [22-24,28-30, 34,37-41,44,46-48,50,51] or aimed at motivating health-promoting behavior within the day to achieve or maintain a specific behavioral target or threshold throughout that day (eg, achieving daily dietary fat goals) [21,25,27,33,35,45]. In only 5 studies [21,25,30,39,45], the feedback was *actionable*, meaning that it incorporated instructions that explicitly communicated *when, where,* and *how* to enact a goal-directed behavior.

#### Feedback Prompting and Delivery Modes

A total of 17 studies [22-24,26,27,29,34,39-42,44,46-48,50,51] used user-initiated feedback, in which the user initiated feedback delivery by providing his or her self-monitoring data; 11 studies [21,25,28,30-33,35-38] used a passive form of feedback prompting that did not require user interaction to initiate feedback delivery; and 3 studies [43,45,49] employed both methods. Nearly all the studies used an automated form of feedback delivery. All but one of the 17 studies that implemented user-initiated feedback relied on self-reported data. A total of 9 studies [22,23,34,39,42,44,46,47,51] based feedback on measures of behavior that were self-reported via diaries or Web-based self-monitoring tools, 4 studies [24,26,27,29] relied on self-reported measures from activity monitors (eg, pedometers); and 4 studies [40,41,48,50] used a combination self-reported dietary intake and self-reported measures from an activity monitor. All 11 studies [21,25,28,30-33,35-38] that employed a passive form of feedback prompting used objectively assessed data. Nine studies [25,28,30,32,33,35-38] used Internet- or Wi-Fi-connected activity monitors, and 2 studies [21,31] used a mandometer, a Bluetooth-connected scale that measures eating rate, or a heart rate monitor. The remaining 3 studies [43,45,49] used a combination of user-initiated and passive feedback methods. One study [45] used a glucometer.

### Efficacy of Feedback Interventions

The studies’ outcomes are described in Multimedia Appendix 5. We determined that 9 studies explicitly tested the use of feedback to motivate behavior change or significantly modulate body weight or body composition or glycated hemoglobin. Of the 9 studies that tested feedback efficacy, 1 was in the area of diet- or nutrition-related behavior [21], 5 focused on PA or sedentary behavior [26,27,30,34,37], and 3 focused on diet and PA or sedentary behavior [39,45,49]. Most of the remaining studies tested a comprehensive intervention in which feedback was implemented as one of the multiple behavior change strategies. Of the 9 studies that tested the efficacy of feedback, 4 had significant findings [21,30,37,45] and 5 did not [26,27,34,39,49].

Regarding feedback content, 3 of the 4 studies with significant findings [21,30,45] and 3 of the 5 studies without significant findings [27,34,39] used both goal- or target-oriented feedback and actionable feedback. In addition, feedback was provided continuously in 3 of the 4 studies with significant findings [21,30,37] and in only 1 of the 5 studies without significant findings [49]. Concerning feedback prompting, 3 of the 4 studies with significant findings used objectively collected data and passive feedback delivery methods [21,30,37], and the fourth [45] used both passive and user-initiated assessment and feedback methods.

### Practical Framework for Just-in-Time Feedback Design

**Figure 2:**
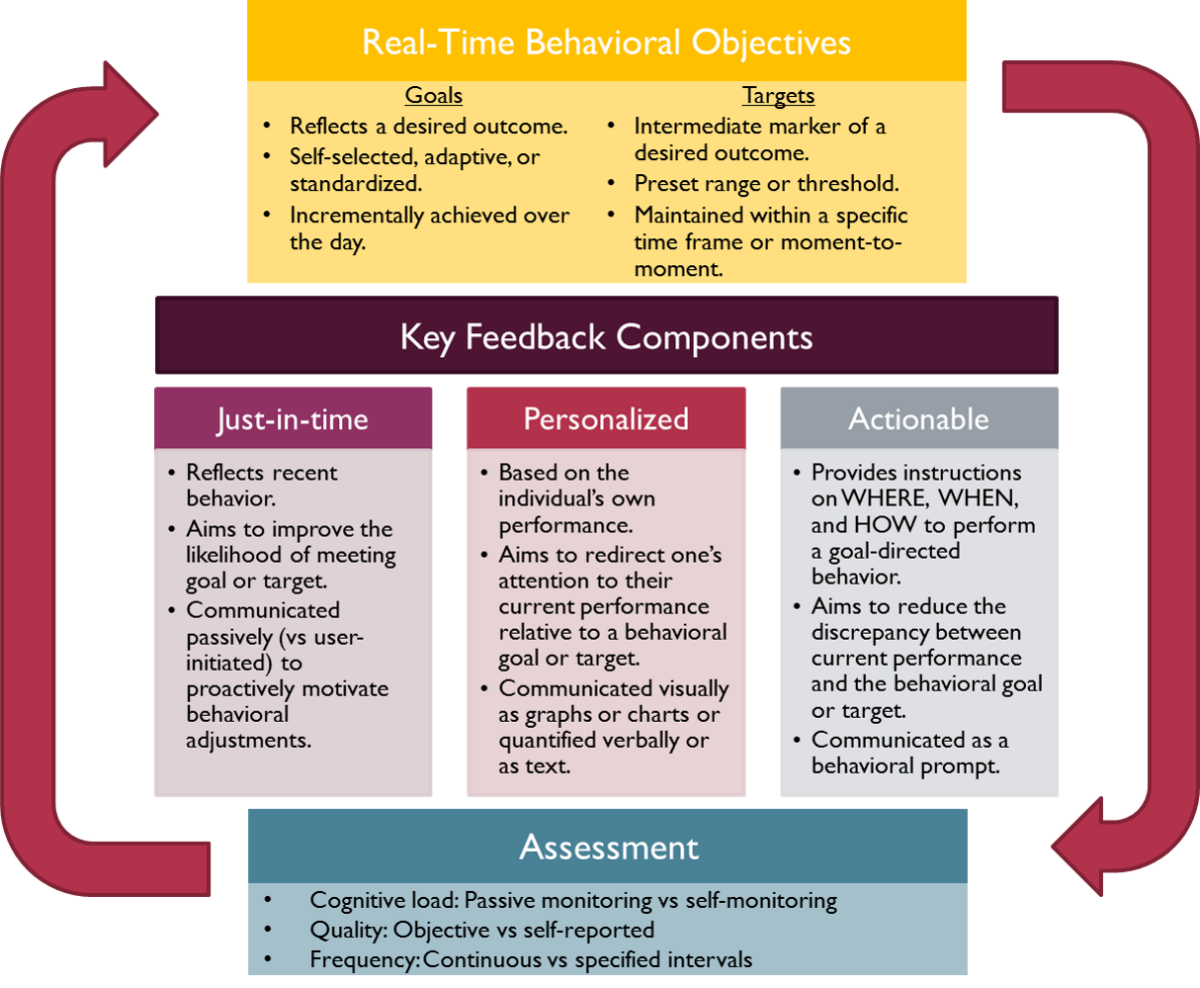
Practical framework for designing just-in-time feedback.

On the basis of the results of our review, we developed a practical framework (Figure 2) that highlights key factors to be considered when developing just-in-time feedback for technology-enhanced diet and PA interventions. We suggest that behavioral objectives (goals or targets) serve as the guiding context for just-in-time feedback and that the selected method of behavioral assessment is the foundation that determines the level of interaction between a user or participant and an external agent or researcher. We propose three characteristics of feedback to take into account: timeliness, personalization, and action orientation.

#### Behavioral Objectives

Most behavioral theories posit that setting a goal is a key behavior change strategy. Behavioral objectives can be framed as goals or targets. Behavioral goals reflect a desired outcome that is achieved incrementally (eg, over a day); can be static (eg, 10,000 steps per day), or adaptive (adjusted incrementally based on performance achievements) and can be self-selected or assigned by an interventionist or health care professional. Comparatively, behavioral targets can be considered intermediate markers or behavioral mediators of achieving a goal that may be explicitly stated or implicitly understood. For example, a behavioral target could be to take a 5-min activity break after sitting for an hour (ie, reduce sedentary behavior) in an effort to achieve a 10,000-steps-per-day goal (ie, increase PA). Thus, behavioral targets are often set at shorter intervals (ie, within the day) than are goals to keep individuals on track for goal attainment. Behavioral targets can also refer to maintaining a continuously assessed marker within a certain predefined range from moment to moment (ie, blood glucose levels) and can be set at moment-to-moment level to achieve an often implicitly understood distal goal (ie, glycemic control). The chosen behavioral objective determines the context for which the feedback content is designed, as well as the appropriate time frame in which it is delivered.

#### Behavioral Assessment

The method used to measure the behavior on which feedback is being provided is an important factor for interventions that incorporate just-in-time feedback. The assessment method determines the cognitive load of self-monitoring, the quality of the data, and the frequency and timeliness with which feedback is delivered. Advancements in wearable sensor technology influence these aspects of data collection, particularly in the area of PA behavior. Newer activity monitors can not only objectively and continuously measure movement (eg, steps) and estimate energy expenditures but also transfer data to another device (eg, mobile phones) or servers wirelessly. This enables the generation and delivery of feedback without any user-initiated input.

#### Timeliness

Just-in-time feedback is defined as providing the right support at the right moment and in the right amount [5,11,12]. In just-in-time adaptive interventions, the right moment might consider the person’s state of vulnerability or opportunity or receptivity. Consistent with the FIT, just-in-time feedback reflects recent behavior and provides guidance at a critical moment when a person’s attention might need to be refocused on the goal-directed behavior. The intent of delivering just-in-time feedback is to proactively motivate behavioral adjustments to limit or reverse the widening of discrepancies between current performance and a behavioral goal or target to facilitate the attainment of that goal or target. As such, just-in-time feedback considers the time frame during which goal achievement is specified to occur. For example, if one has a goal to achieve 10,000 steps per day, just-in-time feedback would be provided before the end of the day to increase the likelihood of achieving that day’s step goal.

#### Personalization

Rather than being generic or group-based, personalized feedback is based on an individual’s own performance and goal. The intent of personalizing feedback is to inform a person about his or her current performance relative to his or her behavioral goal or target (ie, the discrepancy). This message can be communicated visually as graphs or charts (eg, a progress bar) or quantified as text (eg, 3000 more steps to meet your goal) to meet the research needs or the user’s preference.

#### Action Orientation

Feedback that is actionable aims to instruct a patient or participant to engage in behaviors that will improve the likelihood of goal attainment. Action plans are designed to promote small or large behavior changes with a high likelihood of success and should indicate *when, where,* and *how* to enact a goal-directed behavior [54]. Actionable feedback provides behavioral guidance aimed at reducing the discrepancy between current performance and a behavioral goal or target. In just-in-time interventions, action planning varies from its traditional sense of intention formation [54] such that it can be communicated explicitly as instructions for behaviors to be enacted at critical moments [55,56]. These instructions act as behavioral triggers and can be communicated through prompts (eg, Try going for a 30-min walk after dinner tonight to get the 2500 more steps you need to meet your 10,000 step goal today).

## Discussion

### Summary of Key Findings

Our comprehensive literature search yielded 31 studies that met our eligibility criteria. Most of these studies provided feedback that was *just-in-time* (100%, 31/31), *goal-oriented* (75%, 23/31), and *personalized* (97%, 30/31); however, only 5 studies (15%, 5/31) provided *actionable* feedback. Interventions with significant effects featured continuously available and personalized feedback that was actionable or goal-oriented and used objectively assessed data and passively initiated feedback delivery methods. On the basis of these findings, we have proposed a framework for designing just-in-time feedback that incorporates three key content characteristics (timeliness, personalization, and action orientation) relative to a known behavioral goal or target and highlights important considerations regarding the quality and frequency of the behavioral assessment from which just-in-time feedback is derived.

### Comparison With Previous Literature

To our knowledge, our review is unique in that its primary goal was to examine the use of just-in-time feedback in diet and PA interventions. Only one other similar systematic review has been conducted. In that review [17], the authors concluded that the use of personalized feedback was an effective BCT in weight-loss interventions after demonstrating that individuals receiving Internet-delivered personalized feedback lost on average 2 kg more than those receiving no personalized feedback; however, the authors did not analyze the effectiveness of other feedback characteristics. In another review, Michie and colleagues [56] demonstrated through a meta-regression analysis that healthy eating and PA interventions that implemented behavioral monitoring plus one BCT (eg, action planning, prompting specific goal setting, providing feedback on performance, and prompting review of behavioral goals) were more effective than those that did not. However, the authors acknowledged the need to experimentally test the most effective combination of BCTs [56]. Furthermore, only one other known model for developing feedback exists. Hysong and colleagues [57] examined the use of feedback for improving clinical practice guideline adherence and developed the model of actionable feedback. The model posits that an optimal effect on clinical performance can be achieved by providing feedback that is timely, individualized, nonpunitive, and based on customizable performance data. Action was an implied outcome of providing optimal feedback in the model. These previous studies highlight some potentially effective components of feedback that can motivate goal-directed behavior change and that overlap with those put forth in this review.

Another key finding of our review was that only a few of the reviewed studies provided goal-oriented feedback that was actionable. Of the 31 studies included in the review, 23 incorporated behavioral goals; however, only 5 [21,25,30,39,45] gave actionable suggestions on when, where, and how to achieve those goals. In theory, intention precedes action [58]; however, research shows that the intention alone does not often result in actual behavior change [59-61]. Action planning or intention implementation is one strategy to help people transform their intention into action [54]. Traditionally, individuals conceive action plans before acting as their commitment to perform behaviors when opportunities arise [54] and can reevaluate these plans weekly [62]. Such plans often describe where, when, or how frequently to perform the behavior (eg, I intend go to the gym 3 days per week) [55]. Individuals may or may not receive feedback regarding their action plans. In this study *actionable* or *actionable feedback* refers more specifically to information in the form of suggestions an external agent provides to an individual about *where* and *when* to perform the behavior and instructions on *how* to perform the behavior. Examples of actionable feedback in the reviewed studies included multiple, daily behavioral prompts to perform PA breaks of specific durations [25], daily booster messages with exercise prescriptions to achieve the current day’s step goal [30], and just-in-time dietary recommendations (eg, Be aware of high fat snacks tonight) [39]. Our definition of actionable feedback is consistent with Michie and colleagues’ Coventry, Aberdeen, and London-Refined (CALO-RE) taxonomy of BCTs [55]. It is also consistent with the concept of providing supportive information, advice, and feedback at critical moments in just-in-time adaptive interventions [5,11]. This review demonstrates that the provision of actionable feedback in diet and PA interventions is an underutilized behavior change strategy. Relatedly, reviews of seven wearable activity trackers and 40 top-rated diet and PA mobile phone apps found that the integration of BCTs related to action planning and providing instructions consistent with CALO-RE taxonomy was not uncommon [63,64]. However, only two activity trackers helped users identify *where* and *when* to perform PA. Given the increased bidirectional communication capabilities offered by the technology being used to facilitate behavioral interventions, actionable feedback as defined here will likely be more frequently incorporated into interventions as a supportive behavior change strategy. One example of such an intervention is the MyBehavior mobile app. MyBehavior is based on the Fogg behavioral model that applies theoretical principles to technology design by creating tools to prompt low-effort actions that can be triggered even when motivation is low [65]. MyBehavior was designed to generate personalized, actionable insights on when, where, and how to achieve the set goals [66]. The behavioral outcomes from the MyBehavior trial have not yet been published. Future research to determine the efficacy of actionable versus nonactionable feedback as a behavior change strategy will be needed.

### Strengths and Limitations

Our review is strengthened by its focus on key theory-based characteristics of feedback delivered as behavior change interventions. We focused on only diet and PA interventions rather than looking more broadly across additional health behaviors. We did this in part because it is unclear how generalizable our findings might be to other behaviors or health-related outcomes. In addition, studies eligible for inclusion could have included multiple types of feedback, but at least one form of feedback had to meet our definition of just-in-time feedback. We believe this approach strengthens this review by enabling us to make conclusions that facilitate the progress of intervention science into a future in which feedback can be generated and delivered just in time, thereby preparing researchers for continued advancements in technology. Finally, our synthesis of the available data enabled us to develop a framework for designing just-in-time feedback for health behavior change interventions.

Despite these strengths, we were unable to conduct a meta-analysis primarily because of the variability in targeted behavior and study outcomes. Additionally, because it was not clear whether the included studies monitored the delivery, receipt or viewing, or comprehension of the provided feedback, we were not able to conclusively determine the efficacy of using feedback or which feedback feature(s) might be more effective than others. However, we found that feedback was continuously available, goal-oriented, or actionable in 3 of the 4 studies with significant intervention effects. In addition, the sample sizes, intervention durations, and interventions outcomes of studies with significant findings ranged widely. Interventions of longer duration could have been more likely to have significant findings. However, the duration of studies with significant findings was generally shorter than studies with nonsignificant findings (4 weeks to 12 months vs 2 weeks to 24 months). Excluding studies for providing feedback more than 24 hours after a person performed the target behavior limited the number of eligible studies; however, we believe that it was consistent with the advancement of body sensor technology and therefore important to the context of the review. Another important limitation to consider is that most of the included studies did not recruit participants from a representative, diverse population, thus limiting the generalizability of the findings. Finally, the cost of wearable body sensors and wireless devices could be a potential limitation for scaling up the technology-based interventions.

### Implications for Future Research

Before this study, few reviews had critically examined the use of feedback in diet and PA interventions. As advancements in technology continue to improve bidirectional communication between investigators and their participants, optimizing feedback messages will be key to future interventions. The systematic review and the framework we propose represent a foundation for designing feedback messages for future just-in-time diet- or PA-based interventions. Investigators may use the framework to ensure feedback developed for their interventions contain content that is theoretically and empirically supported to have a positive effect on behavior change. However, it is unclear from this review how many of the proposed components are needed to effectively motivate behavior change. Empirical research will be needed to determine the optimal combination of feedback components.

## Appendix

Multimedia Appendix 1 Data extraction form.

Multimedia Appendix 2 Characteristics of included intervention studies.

Multimedia Appendix 3 Risk of bias summaries for included intervention studies.

Multimedia Appendix 4 Descriptions and summaries of feedback features in included intervention studies.

Multimedia Appendix 5 Summaries of outcomes and feedback efficacy for included intervention studies.

## Abbreviations

BCT: behavior change technique
FIT: feedback intervention theory
IG: intervention group
PA: physical activity
PDA: personal digital assistant
PRISMA: Preferred Reporting of Systematic Reviews and Meta-Analyses
SCT: social cognitive theory
