# Supplementary material for: Just-in-Time Feedback in Diet and Physical Activity Interventions: Systematic Review and Practical Design Framework
Source: J Med Internet Res. 2018 Mar 22;20(3):e106. doi: 10.2196/jmir.8701 (PMC5887039; doi:10.2196/jmir.8701)
Supplement: Multimedia Appendix 1 [file jmir_v20i3e106_app1.pdf]

Supplement 1. Data extraction form

**Study Characteristics**					
Intervention type	Diet	Physical activity	Both		
Pilot study	Yes	No			
Country of study					
**Participant Characteristics**					
Study population	General population	Patient population (please specify)	Other population (please specify)		
Age range					
Mean age					
Percent female					
Percent overweight/obese					
# of participants enrolled					
# of participants completed					
**Intervention Characteristics**					
Study design	Randomized control trial	Cohort study	Other (please specify)		
Behavioral theory used					
Intervention duration					
Targeted behavior(s)					
Behavioral assessment methods	Self-reported (please specify)	Objectively measured (please specify)			
Behavioral goal(s)					
**Feedback Characteristics**					
Content (control group)					
Content (intervention group)					
Actionable	Yes	No			
Frequency					
Timing					
Mode of delivery	Mobile phone/SMS text messages	Smartphone apps	Wearable devices (e.g., Fitbit)	Website	Other (please specify)
Prompt	Passive	User-initiated			
**Study Results**					
Within-group changes (separate row for each outcome)	Outcome(s)	Magnitude of change:	Significance of change:		
Between-group differences (separate row for each outcome	Outcome(s)	Magnitude of difference:	Significance of difference:		
