# Supplementary material for: Just-in-Time Feedback in Diet and Physical Activity Interventions: Systematic Review and Practical Design Framework
Source: J Med Internet Res. 2018 Mar 22;20(3):e106. doi: 10.2196/jmir.8701 (PMC5887039; doi:10.2196/jmir.8701)
Supplement: Multimedia Appendix 2 [file jmir_v20i3e106_app2.pdf]

Table 1. Characteristics of diet only intervention studies.

Author, year	N	Sample	Mean age (SD[a]; range), years	Overweight or obese n (%)	Study design, duration	Group
Ford, 2010 [21]	106	Children	12.6 (2.3; 9-17.5)	106 (100)	RCT[b], 12 months	IG[c]: mandometer training<br>CG[d]: standard care
Mummah, 2016 [22]	17	Adults	42.0 (7.3; 18-50)	17 (100)	RCT, 12 weeks	IG: vegethon mobile app<br>CG: waitlist control
Pedersen, 2016 [23]	1488[e]	Children	12.9 (NR[f]; 11-16)	NR	Clustered RCT[g], 11 weeks	IG1: text message feedback<br>IG2: text message feedback + nutrition education<br>CG: minimal intervention control

[a]SD: standard deviation.
[b]RCT: randomized controlled trial.
[c]IG: intervention group.
[d]CG: control group.
[e]Refers to completers or analytical sample.
[f]NR: not reported.
[g]Randomization occurred at a group level (school, medical practice, team, or hospital).

Table 2. Characteristics of PA or sedentary behavior only intervention studies.

Author, year	N	Sample	Mean age (SD[a]; range), years	Overweight or obese n (%)	Study design, duration	Group
Adams, 2013 [24]	20	Inactive adults	36.9 (9.2; 18-65)	20 (100)	RCT[b] (pilot), 6 months	IG[c]1: adaptive goals + performance feedback<br>IG2: static goals + encouraging messages
Bond, 2014 [25]	35	Adults	47.5 (13.5; 21-70)	35 (100)	Within-subjects counter-balanced experimental trial, 3-, 7-day periods	IG1: 3-min activity break after 30 min sedentary<br>IG2: 6-min activity break after 60 min sedentary<br>IG 3: 12-min activity break 120 min sedentary
Chapman, 2016 [26]	64	Adults	45 (NR; 23-69)	NR[e]	RCT, 2 weeks	IG: step tracking + feedback<br>CG[d]: step tracking only
Choi, 2016 [27]	30	Pregnant, inactive women	33.7 (2.6; 18-40)	Not applicable	RCT (pilot), 12 weeks	IG: blinded FitBit + feedback<br>CG: Blinded FitBit

Hooke, 2016 [28]	17	Children in acute lympho-blastic leukemia maintenance	8.7 (3.1; 6-15)	6 (37)	Within-subjects, single-arm intervention, 2 weeks	IG: FitBit + emails
King, 2008 [29]	37	Inactive adults	60.2 (7.2; ≥50)	NR	RCT (pilot), 8 weeks	IG: PDA[f]-based intervention<br>CG: waitlist control
Martin, 2015 [30]	48	Inactive patients from an ambulatory cardiology center	58 (8; 18-69)	26 (54)[i]	RCT with sequential randomization, 4 weeks	IG1: unblinded tracking (phase 1) + texts (phase 2)<br>IG2: unblinded tracking (phase 2) without texts (phase 2)<br>CG: blinded digital activity tracking (phases 1 and 2)
McManus, 2008 [31]	210	Children in 4th and 5th grades	10.4 (0.9; 9-11)	NR	Clustered RCT[g], 4 weeks with 6-month follow-up	IG1: education + feedback school<br>IG2: no education + feedback school<br>CG: control school
Patel, 2016 [32]	288	Adults	41.0 (11.9; ≥50)	NR	Clustered RCT[g], 13 weeks with 13-week follow-up	IG1: comparison to top quartile + incentive<br>IG2: comparison to mean performance + incentive<br>IG3: comparison to top quartile only<br>IG4: comparison to mean performance only
Paul, 2016 [33]	23	Adults with stroke	56 (10; NR)	NR	RCT (pilot), 6 weeks	IG: STARFISH program<br>CG: usual care control
Prestwich, 2016 [34]	124	Inactive adults	23.8 (11.0; NR)	NR	RCT, 2 weeks	IG1: goal setting + self-monitoring + feedback<br>IG2: goal setting + self-monitoring<br>IG3: goal setting only
van der Weegen, 2015 [35]	199	Inactive, adults with type 2 diabetes or chronic obstructive pulmonary disease	57.9 (7.6; 40-70)	NR	Clustered RCT[g], 4-6 months with 3-month follow-up	IG1: self-management support program + activity monitor<br>IG2: self-management support program only

						CG: standard care
van Nimwegen, 2013 [36]	586	Inactive adults with Parkinson's disease	65.5 (7.6; 40-75)	NR	Clustered RCT[g], 24 months	IG: ParkFit program<br>CG: general physiotherapy program
Walsh, 2016 [37]	55	Young adults	20.6 (2.1; 17-26)	NR	RCT (pilot), 5 weeks	IG: pedometer + feedback<br>CG: pedometer only
Wijsman, 2013 [38]	235	Inactive adults	64.8 (40.8; 60-70)	NR	RCT, 13 weeks	IG: DirectLife activity program<br>CG: waitlist control

[a]SD: standard deviation.
[b]RCT: randomized controlled trial.
[c]IG: intervention group.
[d]CG: control group.
[e]NR: not reported.
[f]PDA: personal digital assistant.
[g]Randomization occurred at a group level (school, medical practice, team, or hospital).

Table 3. Characteristics of diet and PA or sedentary behavior intervention studies.

Author, year	N	Sample	Mean age (SD[a]; range), years	Overweight or obese n (%)	Study design, duration	Group
Burke, 2012 [39]	210	Adults	46.8 (9.0; ≤59)	210 (100)	RCT[b], 24 months	IG[c]1: PDA[e]+ feedback<br>IG2: PDA only<br>IG3: paper diary
Collins, 2012 [40]	309	Adults	42 (10.2; 18-60)	309 (100)	RCT, 3 months	IG1: enhanced weight loss<br>IG2: basic weight loss<br>CG[d]: waitlist control
Fassnacht, 2015 [41]	49	Children in 4th grade	9.6 (0.4; 8-10)	14 (29)	RCT (pilot), 8 weeks with 4-week follow-up	IG: short message service (SMS) monitoring + feedback<br>CG: educational sessions only
Fujii, 2009 [42]	650	Employed adults	40.2 (10.1; NR[f])	Men: 144 (36.8)<br>Women: 26 (10.0)	Within-subjects, single-arm intervention, 4 months	IG: computer-based, tailored lifestyle modification support (Health up Navi)
Jakicic, 2016 [43]	471	Adults	30.9 (27.8-33.7[j]; 18-35)	471 (100)	RCT, 24 months	IG1: enhanced program + feedback<br>IG2: standard intervention
Khanna, 2014 [44]	75	Spanish-speaking adults with type 2 diabetes	52.0 (12; NR)	NR	RCT, 12 weeks	IG: automated telephone nutrition support counseling<br>CG: waitlist control with standard care
Lim, 2016 [45]	100	Adults with type	65.1 (5.0; ≥60)	NR	RCT, 6 months	IG1: u-healthcare<br>IG2: self-monitored

		2 diabetes				blood glucose
Nollen, 2014 [46]	51	Low-income, minority girls	11.3 (1.6; 9-14)	30 (59)	RCT (pilot), 12 weeks	IG: mobile technology<br>CG: educational materials only
Sacks, 2009 [47]	811	Adults	52 (9; 30-70)	811 (100)	RCT, 24 months	IG1: low fat, average protein diet<br>IG2: low fat, high protein diet<br>IG3: high fat, average protein diet<br>IG4: high fat, high protein diet
Shapiro, 2008 [48]	58	Children	8.7 (2.3; 5-13)	54 (93)	RCT (pilot), 8 weeks	IG1: SMS + feedback<br>IG2: paper diaries<br>CG: no-monitoring control
Shuger, 2011 [49]	197	Inactive adults	46.9 (10.8; 18-64)	197 (100)	RCT, 9 months	IG1: group-based weight loss program and armband<br>IG2: armband alone<br>IG3: group-based weight loss program alone<br>CG: weight loss program
Steinberg, 2013 [50]	50	Women	38.3 (8.2; 25-50)	50 (100)	RCT (pilot), 6 months	IG: Shape Plan<br>CG: educational materials
Willey, 2016 [51]	10	Adults	43.5 (NR; 30-50)	10 (100)	Within-subjects, single-arm intervention (pilot), 12 weeks	IG: YouPlus Health mobile coaching platform

[a]SD: standard deviation.
[b]RCT: randomized controlled trial.
[c]IG: intervention group.
[d]CG: control group.
[e]PDA: personal digital assistant.
[f]NR: not reported.

References

21. Ford AL, Bergh C, Sodersten P, Sabin MA, Hollinghurst S, Hunt LP, Shield JP. Treatment of childhood obesity by retraining eating behaviour: Randomised controlled trial. BMJ. 2009 Jan 05;340:b5388. PMID: 20051465. doi: 10.1136/bmj.b5388.
22. Mummah SA, Mathur M, King AC, Gardner CD, Sutton S. Mobile technology for vegetable consumption: A randomized controlled pilot study in overweight adults. JMIR mHealth and uHealth. 2016 May 18;4(2):e51. PMID: 27193036. doi: 10.2196/mhealth.5146.
23. Pedersen S, Gronhoj A, Thogersen J. Texting your way to healthier eating? Effects of participating in a feedback intervention using text messaging on adolescents' fruit and vegetable intake. Health Educ Res. 2016 Apr;31(2):171-84. PMID: 26850061. doi: 10.1093/her/cyv104.
24. Adams MA, Sallis JF, Norman GJ, Hovell MF, Hekler EB, Perata E. An adaptive physical activity intervention for overweight adults: A randomized controlled trial. PLoS One. 2013;8(12):e82901. PMID: 24349392. doi: 10.1371/journal.pone.0082901.
25. Bond DS, Thomas JG, Raynor HA, Moon J, Sieling J, Trautvetter J, Leblond T, Wing RR. B-mobile--a smartphone-based intervention to reduce sedentary time in overweight/obese individuals: A within-subjects experimental trial. PLoS One. 2014;9(6):e100821. PMID: 24964010. doi: 10.1371/journal.pone.0100821.
26. Chapman GB, Colby H, Convery K, Coups EJ. Goals and social comparisons promote walking behavior. Med Decis Making. 2016 May;36(4):472-8. PMID: 26139447. doi: 10.1177/0272989X15592156.
27. Choi J, Lee JH, Vittinghoff E, Fukuoka Y. mHealth physical activity intervention: A randomized pilot study in physically inactive pregnant women. Matern Child Health J. 2016 May;20(5):1091-101. PMID: 26649879. doi: 10.1007/s10995-015-1895-7.

28. Hooke MC, Gilchrist L, Tanner L, Hart N, Withycombe JS. Use of a fitness tracker to promote physical activity in children with acute lymphoblastic leukemia. Pediatr Blood Cancer. 2016 Apr;63(4):684-9. PMID: 26756736. doi: 10.1002/pbc.25860.

29. King AC, Ahn DK, Oliveira BM, Atienza AA, Castro CM, Gardner CD. Promoting physical activity through hand-held computer technology. Am J Prev Med. 2008 Feb;34(2):138-42. PMID: 18201644. doi: 10.1016/j.amepre.2007.09.025.

30. Martin SS, Feldman DI, Blumenthal RS, Jones SR, Post WS, McKibben RA, Michos ED, Ndumele CE, Ratchford EV, Coresh J, Blaha MJ. Mactive: A randomized clinical trial of an automated mHealth intervention for physical activity promotion. J Am Heart Assoc. 2015 Nov 09;4(11). PMID: 26553211. doi: 10.1161/JAHA.115.002239.

31. McManus AM, Masters RS, Laukkanen RM, Yu CC, Sit CH, Ling FC. Using heart-rate feedback to increase physical activity in children. Prev Med. 2008 Oct;47(4):402-8. PMID: 18590757. doi: 10.1016/j.ypmed.2008.06.001.

32. Patel MS, Volpp KG, Rosin R, Bellamy SL, Small DS, Fletcher MA, Osman-Koss R, Brady JL, Haff N, Lee SM, Wesby L, Hoffer K, Shuttleworth D, Taylor DH, Hilbert V, Zhu J, Yang L, Wang X, Asch DA. A randomized trial of social comparison feedback and financial incentives to increase physical activity. Am J Health Promot. 2016 Jul;30(6):416-24. PMID: 27422252. doi: 10.1177/0890117116658195.

33. Paul L, Wyke S, Brewster S, Sattar N, Gill JM, Alexander G, Rafferty D, McFadyen AK, Ramsay A, Dybus A. Increasing physical activity in stroke survivors using starfish, an interactive mobile phone application: A pilot study. Top Stroke Rehabil. 2016 Jun;23(3):170-7. PMID: 27077973. doi: 10.1080/10749357.2015.1122266.

34. Prestwich A, Conner M, Hurling R, Ayres K, Morris B. An experimental test of control theory-based interventions for physical activity. Br J Health Psychol. 2016 Nov;21(4):812-26. PMID: 27169809. doi: 10.1111/bjhp.12198.

35. van der Weegen S, Verwey R, Spreeuwenberg M, Tange H, van der Weijden T, de Witte L. It's life! Mobile and web-based monitoring and feedback tool embedded in primary care increases physical activity: A cluster randomized controlled trial. J Med Internet Res. 2015 Jul 24;17(7):e184. PMID: 26209025. doi: 10.2196/jmir.4579.

36. van Nimwegen M, Speelman AD, Overeem S, van de Warrenburg BP, Smulders K, Dontje ML, Borm GF, Backx FJ, Bloem BR, Munneke M, ParkFit Study G. Promotion of physical activity and fitness in sedentary patients with parkinson's disease: Randomised controlled trial. BMJ. 2013 Mar 01;346:f576. PMID: 23457213. doi: 10.1136/bmj.f576.

37. Walsh JC, Corbett T, Hogan M, Duggan J, McNamara A. An mhealth intervention using a smartphone app to increase walking behavior in young adults: A pilot study. JMIR mHealth and uHealth. 2016 Sep 22;4(3):e109. PMID: 27658677. doi: 10.2196/mhealth.5227.

38. Wijsman CA, Westendorp RG, Verhagen EA, Catt M, Slagboom PE, de Craen AJ, Broekhuizen K, van Mechelen W, van Heemst D, van der Ouderaa F, Mooijaart SP. Effects of a web-based intervention on physical activity and metabolism in older adults: Randomized controlled trial. J Med Internet Res. 2013 Nov 06;15(11):e233. PMID: 24195965. doi: 10.2196/jmir.2843.

39. Burke LE, Styn MA, Sereika SM, Conroy MB, Ye L, Glanz K, Sevick MA, Ewing LJ. Using mhealth technology to enhance self-monitoring for weight loss: A randomized trial. Am J Prev Med. 2012 Jul;43(1):20-6. PMID: 22704741. doi: 10.1016/j.amepre.2012.03.016.

40. Collins CE, Morgan PJ, Jones P, Fletcher K, Martin J, Aguiar EJ, Lucas A, Neve MJ, Callister R. A 12-week commercial web-based weight-loss program for overweight and obese adults: Randomized controlled trial comparing basic versus enhanced features. J Med Internet Res. 2012 Apr 25;14(2):e57. PMID: 22555246. doi: 10.2196/jmir.1980.

41. Fassnacht DB, Ali K, Silva C, Goncalves S, Machado PP. Use of text messaging services to promote health behaviors in children. J Nutr Educ Behav. 2015 Jan-Feb;47(1):75-80. PMID: 25282200. doi: 10.1016/j.jneb.2014.08.006.

42. Fujii H, Nakade M, Haruyama Y, Fukuda H, Hashimoto M, Ikuyama T, Kaburagi H, Murai E, Okumura M, Sairenchi T, Muto T. Evaluation of a computer-tailored lifestyle modification support tool for employees in Japan. Ind Health. 2009 Jul;47(3):333-41. PMID: 19531920.
43. Jakicic JM, Davis KK, Rogers RJ, King WC, Marcus MD, Helsel D, Rickman AD, Wahed AS, Belle SH. Effect of wearable technology combined with a lifestyle intervention on long-term weight loss: The IDEA randomized clinical trial. JAMA. 2016 Sep 20;316(11):1161-71. PMID: 27654602. doi: 10.1001/jama.2016.12858.
44. Khanna R, Stoddard PJ, Gonzales EN, Villagran-Flores M, Thomson J, Bayard P, Palos Lucio AG, Schillinger D, Bertozzi S, Gonzales R. An automated telephone nutrition support system for spanish-speaking patients with diabetes. Journal of diabetes science and technology. 2014 Nov;8(6):1115-20. PMID: 25239122. doi: 10.1177/1932296814550186.
45. Lim S, Kang SM, Kim KM, Moon JH, Choi SH, Hwang H, Jung HS, Park KS, Ryu JO, Jang HC. Multifactorial intervention in diabetes care using real-time monitoring and tailored feedback in type 2 diabetes. Acta Diabetol. 2016 Apr;53(2):189-98. PMID: 25936739. doi: 10.1007/s00592-015-0754-8.
46. Nollen NL, Mayo MS, Carlson SE, Rapoff MA, Goggin KJ, Ellerbeck EF. Mobile technology for obesity prevention: A randomized pilot study in racial- and ethnic-minority girls. Am J Prev Med. 2014 Apr;46(4):404-8. PMID: 24650843. doi: 10.1016/j.amepre.2013.12.011.
47. Sacks FM, Bray GA, Carey VJ, Smith SR, Ryan DH, Anton SD, McManus K, Champagne CM, Bishop LM, Laranjo N, Leboff MS, Rood JC, de Jonge L, Greenway FL, Loria CM, Obarzanek E, Williamson DA. Comparison of weight-loss diets with different compositions of fat, protein, and carbohydrates. N Engl J Med. 2009 Feb 26;360(9):859-73. PMID: 19246357. doi: 10.1056/NEJMoa0804748.
48. Shapiro JR, Bauer S, Hamer RM, Kordy H, Ward D, Bulik CM. Use of text messaging for monitoring sugar-sweetened beverages, physical activity, and screen time in children: A

pilot study. J Nutr Educ Behav. 2008 Nov-Dec;40(6):385-91. PMID: 18984496. doi: 10.1016/j.jneb.2007.09.014.

49. Shuger SL, Barry VW, Sui X, McClain A, Hand GA, Wilcox S, Meriwether RA, Hardin JW, Blair SN. Electronic feedback in a diet- and physical activity-based lifestyle intervention for weight loss: A randomized controlled trial. Int J Behav Nutr Phy. 2011 May 18;8:41. PMID: 21592351. doi: 10.1186/1479-5868-8-41.

50. Steinberg DM, Levine EL, Askew S, Foley P, Bennett GG. Daily text messaging for weight control among racial and ethnic minority women: Randomized controlled pilot study. J Med Internet Res. 2013 Nov 18;15(11):e244. PMID: 24246427. doi: 10.2196/jmir.2844.

51. Willey S, Walsh JK. Outcomes of a mobile health coaching platform: 12-week results of a single-arm longitudinal study. JMIR mHealth and uHealth. 2016 Jan 08;4(1):e3. PMID: 26747611. doi: 10.2196/mhealth.4933.
