# Supplementary material for: Just-in-Time Feedback in Diet and Physical Activity Interventions: Systematic Review and Practical Design Framework
Source: J Med Internet Res. 2018 Mar 22;20(3):e106. doi: 10.2196/jmir.8701 (PMC5887039; doi:10.2196/jmir.8701)
Supplement: Multimedia Appendix 3 [file jmir_v20i3e106_app3.pdf]

Table 1. Risk of bias summary for diet only intervention studies.

Author, year	Selection bias	Study design	Confounders	Blinding	Data collection method	Withdrawals and dropouts	Global rating
Ford, 2010 [21]	Moderate	Strong	Strong	Moderate	Weak	Strong	Moderate
Mummah, 2016 [22]	Weak	Strong	Strong	Strong	Strong	Moderate	Moderate
Pedersen, 2015 [23]	Strong	Strong	Strong	Moderate	Moderate	Weak	Moderate

Table 2. Risk of bias summary for PA or sedentary behavior only intervention studies.

Author, year	Selection bias	Study design	Confounders	Blinding	Data collection method	Withdrawals and dropouts	Global rating
Adams, 2013 [24]	Weak	Strong	Strong	Weak	Strong	Strong	Weak
Bond, 2014 [25]	Weak	Moderate	N/A[a]	Moderate	Strong	Strong	Moderate
Chapman, 2016 [26]	Weak	Strong	Weak	Moderate	Strong	Strong	Weak
Choi, 2015 [27]	Moderate	Strong	Strong	Weak	Strong	Strong	Moderate
Hooke, 2016 [28]	Moderate	Moderate	N/A	Moderate	Strong	Strong	Strong
King, 2008 [29]	Weak	Strong	Strong	Moderate	Strong	Strong	Moderate
Martin, 2015 [30]	Moderate	Strong	Strong	Moderate	Strong	Strong	Strong
McManus, 2008 [31]	Moderate	Strong	Strong	Moderate	Strong	Strong	Strong
Patel, 2016 [32]	Weak	Strong	Strong	Moderate	Strong	Strong	Moderate
Paul, 2016 [33]	Weak	Strong	Strong	Weak	Strong	Strong	Weak
Prestwich, 2016 [34]	Weak	Strong	Strong	Moderate	Strong	Strong	Moderate
van der Weegen, 2015 [35]	Weak	Strong	Strong	Moderate	Strong	Strong	Moderate
van Nimwegen 2013 [36]	Weak	Strong	Strong	Strong	Strong	Strong	Moderate
Walsh, 2016 [37]	Weak	Strong	Strong	Moderate	Weak	Strong	Weak
Wijsman, 2013 [38]	Weak	Strong	Strong	Weak	Strong	Strong	Weak

Table 3. Risk of bias summary for diet and PA or sedentary behavior intervention studies.

Author, year	Selection bias	Study design	Confounders	Blinding	Data collection method	Withdrawals and dropouts	Global rating
Burke, 2012 [39]	Weak	Strong	Strong	Moderate	Strong	Strong	Moderate
Collins, 2012 [40]	Weak	Strong	Strong	Strong	Moderate	Moderate	Moderate
Fassnacht, 2015 [41]	Moderate	Strong	Strong	Moderate	Weak	Weak	Weak
Fujii, 2009 [42]	Weak	Moderate	N/A[a]	Moderate	Weak	Weak	Weak
Jakicic, 2016 [43]	Weak	Strong	Strong	Strong	Strong	Moderate	Moderate
Khanna, 2014 [44]	Moderate	Strong	Strong	Moderate	Weak	Moderate	Moderate
Lim, 2015 [45]	Moderate	Strong	Strong	Moderate	Strong	Strong	Strong
Nollen, 2014 [46]	Weak	Strong	Weak	Moderate	Strong	Moderate	Weak
Sacks, 2009 [47]	Weak	Strong	Strong	Strong	Strong	Strong	Moderate
Shapiro, 2008 [48]	Weak	Strong	Strong	Moderate	Weak	Weak	Weak
Shuger, 2011 [49]	Weak	Strong	Strong	Moderate	Strong	Moderate	Moderate
Steinberg, 2013 [50]	Weak	Strong	Strong	Moderate	Moderate	Strong	Moderate
Willey, 2016 [51]	Weak	Moderate	N/A	Moderate	Strong	Strong	Moderate

[a]N/A: not applicable (because of study design).

References

21. Ford AL, Bergh C, Sodersten P, Sabin MA, Hollinghurst S, Hunt LP, Shield JP. Treatment of childhood obesity by retraining eating behaviour: Randomised controlled trial. BMJ. 2009 Jan 05;340:b5388. PMID: 20051465. doi: 10.1136/bmj.b5388.

22. Mummah SA, Mathur M, King AC, Gardner CD, Sutton S. Mobile technology for vegetable consumption: A randomized controlled pilot study in overweight adults. JMIR mHealth and uHealth. 2016 May 18;4(2):e51. PMID: 27193036. doi: 10.2196/mhealth.5146.

23. Pedersen S, Gronhoj A, Thogersen J. Texting your way to healthier eating? Effects of participating in a feedback intervention using text messaging on adolescents' fruit and vegetable intake. Health Educ Res. 2016 Apr;31(2):171-84. PMID: 26850061. doi: 10.1093/her/cyv104.

24. Adams MA, Sallis JF, Norman GJ, Hovell MF, Hekler EB, Perata E. An adaptive physical activity intervention for overweight adults: A randomized controlled trial. PLoS One. 2013;8(12):e82901. PMID: 24349392. doi: 10.1371/journal.pone.0082901.

25. Bond DS, Thomas JG, Raynor HA, Moon J, Sieling J, Trautvetter J, Leblond T, Wing RR. B-mobile--a smartphone-based intervention to reduce sedentary time in overweight/obese individuals: A within-subjects experimental trial. PLoS One. 2014;9(6):e100821. PMID: 24964010. doi: 10.1371/journal.pone.0100821.

26. Chapman GB, Colby H, Convery K, Coups EJ. Goals and social comparisons promote walking behavior. Med Decis Making. 2016 May;36(4):472-8. PMID: 26139447. doi: 10.1177/0272989X15592156.

27. Choi J, Lee JH, Vittinghoff E, Fukuoka Y. mHealth physical activity intervention: A randomized pilot study in physically inactive pregnant women. Matern Child Health J. 2016 May;20(5):1091-101. PMID: 26649879. doi: 10.1007/s10995-015-1895-7.

28. Hooke MC, Gilchrist L, Tanner L, Hart N, Withycombe JS. Use of a fitness tracker to promote physical activity in children with acute lymphoblastic leukemia. Pediatr Blood Cancer. 2016 Apr;63(4):684-9. PMID: 26756736. doi: 10.1002/pbc.25860.
29. King AC, Ahn DK, Oliveira BM, Atienza AA, Castro CM, Gardner CD. Promoting physical activity through hand-held computer technology. Am J Prev Med. 2008 Feb;34(2):138-42. PMID: 18201644. doi: 10.1016/j.amepre.2007.09.025.
30. Martin SS, Feldman DI, Blumenthal RS, Jones SR, Post WS, McKibben RA, Michos ED, Ndumele CE, Ratchford EV, Coresh J, Blaha MJ. Mactive: A randomized clinical trial of an automated mHealth intervention for physical activity promotion. J Am Heart Assoc. 2015 Nov 09;4(11). PMID: 26553211. doi: 10.1161/JAHA.115.002239.
31. McManus AM, Masters RS, Laukkanen RM, Yu CC, Sit CH, Ling FC. Using heart-rate feedback to increase physical activity in children. Prev Med. 2008 Oct;47(4):402-8. PMID: 18590757. doi: 10.1016/j.ypmed.2008.06.001.
32. Patel MS, Volpp KG, Rosin R, Bellamy SL, Small DS, Fletcher MA, Osman-Koss R, Brady JL, Haff N, Lee SM, Wesby L, Hoffer K, Shuttleworth D, Taylor DH, Hilbert V, Zhu J, Yang L, Wang X, Asch DA. A randomized trial of social comparison feedback and financial incentives to increase physical activity. Am J Health Promot. 2016 Jul;30(6):416-24. PMID: 27422252. doi: 10.1177/0890117116658195.
33. Paul L, Wyke S, Brewster S, Sattar N, Gill JM, Alexander G, Rafferty D, McFadyen AK, Ramsay A, Dybus A. Increasing physical activity in stroke survivors using starfish, an interactive mobile phone application: A pilot study. Top Stroke Rehabil. 2016 Jun;23(3):170-7. PMID: 27077973. doi: 10.1080/10749357.2015.1122266.
34. Prestwich A, Conner M, Hurling R, Ayres K, Morris B. An experimental test of control theory-based interventions for physical activity. Br J Health Psychol. 2016 Nov;21(4):812-26. PMID: 27169809. doi: 10.1111/bjhp.12198.

35. van der Weegen S, Verwey R, Spreeuwenberg M, Tange H, van der Weijden T, de Witte L. It's life! Mobile and web-based monitoring and feedback tool embedded in primary care increases physical activity: A cluster randomized controlled trial. J Med Internet Res. 2015 Jul 24;17(7):e184. PMID: 26209025. doi: 10.2196/jmir.4579.

36. van Nimwegen M, Speelman AD, Overeem S, van de Warrenburg BP, Smulders K, Dontje ML, Borm GF, Backx FJ, Bloem BR, Munneke M, ParkFit Study G. Promotion of physical activity and fitness in sedentary patients with parkinson's disease: Randomised controlled trial. BMJ. 2013 Mar 01;346:f576. PMID: 23457213. doi: 10.1136/bmj.f576.

37. Walsh JC, Corbett T, Hogan M, Duggan J, McNamara A. An mhealth intervention using a smartphone app to increase walking behavior in young adults: A pilot study. JMIR mHealth and uHealth. 2016 Sep 22;4(3):e109. PMID: 27658677. doi: 10.2196/mhealth.5227.

38. Wijsman CA, Westendorp RG, Verhagen EA, Catt M, Slagboom PE, de Craen AJ, Broekhuizen K, van Mechelen W, van Heemst D, van der Ouderaa F, Mooijaart SP. Effects of a web-based intervention on physical activity and metabolism in older adults: Randomized controlled trial. J Med Internet Res. 2013 Nov 06;15(11):e233. PMID: 24195965. doi: 10.2196/jmir.2843.

39. Burke LE, Styn MA, Sereika SM, Conroy MB, Ye L, Glanz K, Sevick MA, Ewing LJ. Using mhealth technology to enhance self-monitoring for weight loss: A randomized trial. Am J Prev Med. 2012 Jul;43(1):20-6. PMID: 22704741. doi: 10.1016/j.amepre.2012.03.016.

40. Collins CE, Morgan PJ, Jones P, Fletcher K, Martin J, Aguiar EJ, Lucas A, Neve MJ, Callister R. A 12-week commercial web-based weight-loss program for overweight and obese adults: Randomized controlled trial comparing basic versus enhanced features. J Med Internet Res. 2012 Apr 25;14(2):e57. PMID: 22555246. doi: 10.2196/jmir.1980.

41. Fassnacht DB, Ali K, Silva C, Goncalves S, Machado PP. Use of text messaging services to promote health behaviors in children. J Nutr Educ Behav. 2015 Jan-Feb;47(1):75-80. PMID: 25282200. doi: 10.1016/j.jneb.2014.08.006.

42. Fujii H, Nakade M, Haruyama Y, Fukuda H, Hashimoto M, Ikuyama T, Kaburagi H, Murai E, Okumura M, Sairenchi T, Muto T. Evaluation of a computer-tailored lifestyle modification support tool for employees in Japan. Ind Health. 2009 Jul;47(3):333-41. PMID: 19531920.

43. Jakicic JM, Davis KK, Rogers RJ, King WC, Marcus MD, Helsel D, Rickman AD, Wahed AS, Belle SH. Effect of wearable technology combined with a lifestyle intervention on long-term weight loss: The IDEA randomized clinical trial. JAMA. 2016 Sep 20;316(11):1161-71. PMID: 27654602. doi: 10.1001/jama.2016.12858.

44. Khanna R, Stoddard PJ, Gonzales EN, Villagran-Flores M, Thomson J, Bayard P, Palos Lucio AG, Schillinger D, Bertozzi S, Gonzales R. An automated telephone nutrition support system for spanish-speaking patients with diabetes. Journal of diabetes science and technology. 2014 Nov;8(6):1115-20. PMID: 25239122. doi: 10.1177/1932296814550186.

45. Lim S, Kang SM, Kim KM, Moon JH, Choi SH, Hwang H, Jung HS, Park KS, Ryu JO, Jang HC. Multifactorial intervention in diabetes care using real-time monitoring and tailored feedback in type 2 diabetes. Acta Diabetol. 2016 Apr;53(2):189-98. PMID: 25936739. doi: 10.1007/s00592-015-0754-8.

46. Nollen NL, Mayo MS, Carlson SE, Rapoff MA, Goggin KJ, Ellerbeck EF. Mobile technology for obesity prevention: A randomized pilot study in racial- and ethnic-minority girls. Am J Prev Med. 2014 Apr;46(4):404-8. PMID: 24650843. doi: 10.1016/j.amepre.2013.12.011.

47. Sacks FM, Bray GA, Carey VJ, Smith SR, Ryan DH, Anton SD, McManus K, Champagne CM, Bishop LM, Laranjo N, Leboff MS, Rood JC, de Jonge L, Greenway FL, Loria CM, Obarzanek E, Williamson DA. Comparison of weight-loss diets with different compositions of fat, protein, and carbohydrates. N Engl J Med. 2009 Feb 26;360(9):859-73. PMID: 19246357. doi: 10.1056/NEJMoa0804748.

48. Shapiro JR, Bauer S, Hamer RM, Kordy H, Ward D, Bulik CM. Use of text messaging for monitoring sugar-sweetened beverages, physical activity, and screen time in children: A

pilot study. J Nutr Educ Behav. 2008 Nov-Dec;40(6):385-91. PMID: 18984496. doi: 10.1016/j.jneb.2007.09.014.

49. Shuger SL, Barry VW, Sui X, McClain A, Hand GA, Wilcox S, Meriwether RA, Hardin JW, Blair SN. Electronic feedback in a diet- and physical activity-based lifestyle intervention for weight loss: A randomized controlled trial. Int J Behav Nutr Phy. 2011 May 18;8:41. PMID: 21592351. doi: 10.1186/1479-5868-8-41.

50. Steinberg DM, Levine EL, Askew S, Foley P, Bennett GG. Daily text messaging for weight control among racial and ethnic minority women: Randomized controlled pilot study. J Med Internet Res. 2013 Nov 18;15(11):e244. PMID: 24246427. doi: 10.2196/jmir.2844.

51. Willey S, Walsh JK. Outcomes of a mobile health coaching platform: 12-week results of a single-arm longitudinal study. JMIR mHealth and uHealth. 2016 Jan 08;4(1):e3. PMID: 26747611. doi: 10.2196/mhealth.4933.
