# Supplementary material for: Just-in-Time Feedback in Diet and Physical Activity Interventions: Systematic Review and Practical Design Framework
Source: J Med Internet Res. 2018 Mar 22;20(3):e106. doi: 10.2196/jmir.8701 (PMC5887039; doi:10.2196/jmir.8701)
Supplement: Multimedia Appendix 4 [file jmir_v20i3e106_app4.pdf]

Table 1. Description and summary of feedback features in diet only intervention studies.

Author, Year	Targeted behavior[a], monitoring method[b]	Behavioral target or goal[c]	Feedback content	Feedback frequency and timing	Feedback delivery mode	Feedback prompt
Ford, 2010 [21]	Eating speed, mandometer	Intervention group (IG): eat at a speed that matches a recommended rate displayed on the device<br><br>Control group (CG): self-selected dietary goals and encouragement to be active 60 min per day	IG: real-time visual graphic of decreasing plate weight as the user eats<br><br>CG: none specified	Continuously available; with use at evening meals	Mandometer	Passive
Mummah, 2016 [22]	Vegetable intake, mobile app–based self-report	IG: self-select goal from a range of daily vegetable servings (1-10 servings per day)<br><br>CG: none specified	IG: graphical representation of progress toward goals by day, past 7 days, and past 7 weeks<br><br>CG: none specified	Daily; immediate upon self-reported data entry	Mobile phone app	User-initiated
Pedersen, 2016 [23]	Fruit and vegetable (F/V) intake, self-report	IG1/IG2: weekly goals set by participants and adjusted every other week<br><br>CG: none specified	IG1/IG2: F/V intake relative to the self-set weekly goal<br><br>CG: none specified	Nightly; immediate upon self-reported data entry	SMS text messages (short message service, SMS)	User-initiated

Table 2. Description and summary of feedback features in PA or sedentary behavior only intervention studies.

Author, Year	Targeted behavior[a], monitoring method[b]	Behavioral target or goal[c]	Feedback content	Feedback frequency and timing	Feedback delivery mode	Feedback prompt
Adams, 2013 [24]	Steps per day, pedometer	All groups: 10,000 steps per day or adaptive goals based on a rank-order percentile algorithm set for each day	IG1: praise for steps taken or having met daily goals<br><br>IG2: adapted step goal action plan for next day, and praise for steps taken or having met current day's goals.	Daily; within 2 hours of self-reported data entry	Choice of email or SMS text message	User-initiated

Bond, 2014 [25]	Time spent in sedentary behavior, multi-sensor monitor	All groups: static goals of taking 3-6-, or 12-min PA break after 30, 60, or 120 continuous sedentary min	All groups: sedentary min, active min, time until next activity break, just-in-time activity break prompts, and encouragement for performing an activity break	Continuously available (mobile phone–enabled)	Mobile phone app	Passive
Chapman, 2016 [26]	Steps per day, self-reported from a pedometer	None specified	IG: social comparison feedback that corresponded their performance relative to a reference population<br><br>CG: None specified	(1) Daily; immediate upon self-reported data entry and (2) Twice-weekly 3-day summaries	(1) Study-specific website and (2) Email	User-initiated
Choi, 2016 [27]	Steps per day, activity tracker	All groups: Incrementally increase steps per day each week until at least 8500 steps per day for 5+ days per week has been reached	IG: (1) actionable messages aimed at encouraging daily activity, (2) messages regarding progress toward step goals, and (3) graph of the week's daily steps<br><br>CG: none specified	(1) Daily; within-day action plan in response to answered activity prompts (2) Daily; immediate upon self-reported data entry (3) Weekly; based on self-reported data entry	Mobile phone app	User-initiated
Hooke, 2016 [28]	Steps per day, activity tracker	Increasing steps per day by 15–20% weekly up to a 2,000-step increase to reach goals of 12,000 or 15,000 steps per day (girls/boys)	FitBit screenshots of previous day's steps walked and activity levels	Daily	Email	Passive
King, 2008 [29]	Steps per day, self-reported from a pedometer	IG: personalized PA goals<br><br>CG: none specified	IG: cumulative reports of PA min using text and graphic formats<br><br>CG: none specified	(1) Daily; immediate upon self-reported data entry and (2) Weekly cumulative based on self-reported exercise min	PDA program designed specifically for this study	User-initiated
Martin, 2015 [30]	Steps per day, activity tracker	All groups: static goal of 10,000 steps per day	IG1/IG2: (1) daily step count, activity time, and aerobic activity and (2) step count with positive	(1) Continuously available (2) 3X daily (via smart test), customized to	(1) Mobile phone app and Web interfaces and (2) SMS text	Passive

			reinforcement if daily goal was reached, booster messages if the goal was not met<br><br>CG: None specified	participant's wake time, lunch time, and beginning of evening leisure time	messages	
McManus, 2008 [31]	Intensity of physical activity (PA), heart-rate monitor	All groups: static goal of accumulating 30 to 60 min of Moderate-to-vigorous physical activity (MVPA) per day	IG1/IG2: graphical display of heart rate and time spent in the different activity zones (2 weeks of intervention only)<br><br>CG: none specified	Continuously available (via heart rate monitor)	Commercially available heart rate monitor	Passive
Patel, 2016 [32]	Steps per day, mobile phone accelerometer linked to the *Moves* mobile app	All groups: 7000 steps per day	All groups: average number of steps from the previous day per team member with and without comparison to mean performance (50th percentile) or the top quartile (75th percentile) in their IG	Daily	Self-selected from email, SMS text message, or both	Passive
Paul, 2016 [33]	Steps per day, mobile phone accelerometer	IG: personalized week 1 goal of baseline steps per day + increases of 10% and 5% steps from baseline per day thereafter when goals were met<br><br>CG: none specified	IG: step count progress of self and three other virtual group members<br><br>CG: none specified	Continuously available (mobile phone–enabled)	Mobile phone app	Passive
Prestwich, 2016 [34]	Exercise min and intensity, daily Web-based diary	All groups: 600 activity units per week or 30 min of moderate PA 5 times per week	IG1: graphs showing progress toward the weekly goal based on diary entries<br><br>IG2 or IG3: none specified	Nightly; immediate upon self-reported entry	Website designed specifically for this study	User-initiated
van der Weegen, 2015 [35]	Exercise, accelerometer	All groups: 30 min of MVPA on 5+ days per week	IG1: graph of real-time results and history in min of activity with MVPA threshold indicated<br><br>IG2 or CG: none specified	Continuously available (accelerometer-enabled)	Mobile phone app and website designed specifically for this study	Passive

van Nimwegen, 2013 [36]	PA, ambulatory activity monitor	IG: personalized activity objectives (provided at coaching sessions) to achieve 6-month goals<br><br>CG: none specified	IG: motivational strategies with visual feedback about the level of PA during the day<br><br>CG: none specified	Continuously available (activity monitor–enabled)	Ambulatory monitor	Passive
Walsh, 2016 [37]	Steps per day, mobile phone app–connected pedometer	All groups: 30 min of activity or walking daily	IG: steps relative to a goal, as well as distance, time, speed, and calories burned<br><br>CG: None specified	Continuously available (mobile phone–enabled)	Mobile phone app	Passive
Wijsman, 2013 [38]	PA, ankle- and wrist-worn triaxial accelerometer	IG: personalized activity goals based on current activity level and increased weekly<br><br>CG: none specified	IG: (1) visual display of progress toward daily goal and graphs showing activity levels and goal tracking and (2) ersonal e-coach to provide regular updates of PA status and tips to increase activity<br><br>CG: none specified	(1) Immediate; when device was connected to a computer and (2) Upon user request	(1) Website accompanying the commercially available device and (2) Email	Passive

Table 3. Description and summary of feedback features in diet and PA or sedentary behavior intervention studies.

Author, Year	Targeted behavior[a], monitoring method[b]	Behavioral target or goal[c]	Feedback content	Feedback frequency and timing	Feedback delivery mode	Feedback prompt
Burke, 2012 [39]	Energy and dietary fat intake, self-report<br><br>Exercise, PDA with self-monitoring software or standard paper diaries	All groups: (1) personalized calorie intake goals (based on weight and gender) and ≤25% dietary fat intake and (2) exercise goal of 180 min per week by 6 months and 30 min per week increases semiannually thereafter	IG1: (1) just-in-time motivational messages to achieve daily diet goals and (2) 2-day PA action plans to meet weekly goals<br><br>IG2 or IG3: none specified	(1) Daily; at 1 of 4 randomly selected times (2) Every other day; at 1 of 2 selected times	PDA software designed specifically for this study.	User-initiated

Collins, 2012 [40]	Energy intake and exercise, self-reported by Web-based diary<br><br>Steps per day, pedometer	All groups: personalized daily calorie targets to achieve a weight loss of 0.5 to 1 kg per week	IG1: (1) calculations of energy balance and nutrition summary compared with recommended nutrient targets and (2) e-feedback based on diet and PA diary entries, weight loss success, and website usage patterns<br><br>IG2: (1) calculations of energy balance and nutrition summary compared with recommended nutrient targets<br><br>CG: none specified	(1) Daily and weekly (2) Weekly, all based on self-reported diet and PA diary entries	Commercially available website	User-initiated
Fassnacht, 2015 [41]	F/V intake, PA, and screen time, self-reported with parental assistance<br><br>Steps (IG only), pedometer	All groups: static goals of five portions of fruits or vegetables per day, 10,000 steps per day, and <60 min per day screen time	IG: tailored messages based on how many of the three goals were met and improvement or deterioration from the previous day<br><br>CG: none specified	Daily; immediate upon transfer of data to the SMS system	SMS text messages	User initiated
Fujii, 2009 [42]	Behavioral determinants of low salt and low energy intake, F/V intake, healthy eating, and regular exercise by self-report	Self-determined, tangible goals to change diet and exercise	Graphic forms of data records	Daily; immediate upon self-reported data entry	Commercially available web-based computer program	User-initiated
Jakicic, 2016 [43]	MVPA and dietary intake, Web-based diaries or accelerometer, and accompanying Web-based	All groups: personalized, 1200 to 1800 kcal per day calorie goal; 20 to 30% dietary fat intake; 100 min per week of MVPA that increased	IG1: (1) real-time summary of energy expenditure and total MVPA, (2) MVPA in durations of 10 min or longer, and (3) dietary and PA feedback	(1) Continuously available (via device display), (2) Real-time, continuous (via Web access), and (3) Weekly	(1) FIT core device, (2) FIT core website, and (3) Telephone	Passive and user-initiated

	software (intervention only)	every 4 weeks until 300 min per week was achieved	IG2: intervention staff provided feedback on diet and PA diaries	(months 1-6) and monthly (months 7-12)		
Khanna, 2014 [44]	Low glycemic food intake, self-report	IG: substitute low-glycemic index foods for high-glycemic index foods<br><br>CG: none specified	IG: self-assessment with automated voice message based on self-reported dietary intake: positive feedback if <3 servings of high glycemic foods, cautious message if 3 to 4 servings, educational message if >4 servings<br><br>CG: none specified	Daily; immediate upon self-report entry	Telephone (interactive voice response)	User-initiated
Lim, 2016 [45]	Dietary intake, Web-based self-report<br><br>PA, activity monitor<br><br>Glucose monitoring, glucometer (self-monitoring group) or Internet-connected glucometer	All groups: static goals of maintaining or increasing daily activity to >30 min at least 3 times per week, and measuring blood glucose levels at least 8 times per week	IG1: (1) actionable feedback in response to glucometer data aimed to raise glucose levels if <50 mg/dl and lower glucose levels if ≥140 mg/dl, (2) average glucose level, (3) time spent being physically active and calorie expenditure, and (4) total caloric intake and carbohydrate, protein, and fat components<br><br>IG2: None specified	(1) Within 1 min upon glucometer use. (2) Weekly at 10 AM on Mondays and monthly at 11 AM on the last day of the month. (3) Upon self-reported data entry, and (4) Automatically displayed upon data entry	(1) SMS text message, (2) SMS text message, (3) Website designed specifically for this study, and (4) SMS Text message	Passive and user-initiated
Nollen, 2014 [46]	F/V intake (weeks 1-4), sugar-sweetened beverages (SSBs; weeks 5-8), and screen time (weeks 9-12) by	IG: two daily goals related to the targeted behavior<br><br>CG: none specified	IG: progress toward goals and feedback and reinforcement on goal attainment.<br><br>CG: none specified	Daily at 5 preselected times	PDA program designed specifically for this study	User-initiated

	self-monitoring					
Sacks, 2009 [47]	Macronutrient intake, daily food diary, and Web-based self-monitoring tool<br><br>Exercise, Web-based diary	All groups: prescribed macronutrient goals by group, reduced calorie intake (−750 kcal per day deficit), and 90 min moderate exercise per week to achieve a weight loss of 1.5 lbs per week	All groups: graphs (percentage of deviation from goal) about dietary and exercise adherence, and changes in body weight	Daily; immediate upon self-reported data entry	Web-based computerized tracking system designed specifically for this study	User-initiated
Shapiro, 2008 [48]	SSB intake, self-report.<br><br>PA and screen time, self-report in all groups and by pedometer in the IGs	All groups: static goals of 5000 steps, <60 min total screen time, and 0 SSB per day	IG1: (1) number of goals met and evaluation of improvement or deterioration from the previous day<br><br>IG2: (2) self-monitoring feedback<br><br>CG: none specified	(1) Daily; immediate upon self-reported data entry and (2) Weekly	(1) SMS text messages and (2) Verbal	User-initiated
Shuger, 2011 [49]	Daily energy intake, Web-based self-report<br><br>Energy expenditure, armband	All groups: weight loss by healthy lifestyle	IG1 or IG2: (1) cumulative energy expenditure, cumulative MVPA min, steps per day and (2) energy balance<br><br>IG3 or CG: none specified	(1) Continuously available (armband-enabled) and (2) Immediate; when armband and diet data were uploaded to the website	(1) Armband and (2) Commercially available website	Passive and user-initiated
Steinberg, 2013 [50]	SSB and F/V intake, self-report<br><br>Steps per day, self-reported from a pedometer	IG: no SSB, 5+ F/V, an adaptive goal of at least 7000 steps per day increased to up to 10,000 steps per day based on performance<br><br>CG: 10,000 steps per day	IG: (1) summary score of goal attainment with feedback based on the previous day, specific tips if goals were not met and (2) progress summary of goal attainment with graph of progress<br><br>CG: none	(1) Daily; immediate upon self-reported data entry and (2) Weekly (for those with 3+ days of self-monitoring data)	SMS text messages	User-initiated

			specified.			
Willey, 2016 [51]	Diet and activity, app-based self-report	Eat a target ratio of protein to carbohydrates daily and complete three sessions each of core cardiovascular and resistance exercise every 7 to 10 days	(1) Graphical representation of weekly progress toward goal and (2) Motivational messages and advice based on answers to in-app questions about diet and exercise behaviors	(1) Daily; immediate upon self-reported data entry and (2) Daily; immediate upon self-reported data entry (diet) or every 7 to 10 days per when an exercise level was completed (exercise)	Mobile phone app	User-initiated

[a]Refers to the behavior(s) for which feedback was provided.
[b]Refers to the assessment method for the targeted behaviors on which feedback was based.
[c]Refers to goal(s) set for the targeted behavior(s) only.

References

21. Ford AL, Bergh C, Sodersten P, Sabin MA, Hollinghurst S, Hunt LP, Shield JP. Treatment of childhood obesity by retraining eating behaviour: Randomised controlled trial. BMJ. 2009 Jan 05;340:b5388. PMID: 20051465. doi: 10.1136/bmj.b5388.

22. Mummah SA, Mathur M, King AC, Gardner CD, Sutton S. Mobile technology for vegetable consumption: A randomized controlled pilot study in overweight adults. JMIR mHealth and uHealth. 2016 May 18;4(2):e51. PMID: 27193036. doi: 10.2196/mhealth.5146.

23. Pedersen S, Gronhoj A, Thogersen J. Texting your way to healthier eating? Effects of participating in a feedback intervention using text messaging on adolescents' fruit and vegetable intake. Health Educ Res. 2016 Apr;31(2):171-84. PMID: 26850061. doi: 10.1093/her/cyv104.

24. Adams MA, Sallis JF, Norman GJ, Hovell MF, Hekler EB, Perata E. An adaptive physical activity intervention for overweight adults: A randomized controlled trial. PLoS One. 2013;8(12):e82901. PMID: 24349392. doi: 10.1371/journal.pone.0082901.

25. Bond DS, Thomas JG, Raynor HA, Moon J, Sieling J, Trautvetter J, Leblond T, Wing RR. B-mobile--a smartphone-based intervention to reduce sedentary time in overweight/obese individuals: A within-subjects experimental trial. PLoS One. 2014;9(6):e100821. PMID: 24964010. doi: 10.1371/journal.pone.0100821.

26. Chapman GB, Colby H, Convery K, Coups EJ. Goals and social comparisons promote walking behavior. Med Decis Making. 2016 May;36(4):472-8. PMID: 26139447. doi: 10.1177/0272989X15592156.

27. Choi J, Lee JH, Vittinghoff E, Fukuoka Y. mHealth physical activity intervention: A randomized pilot study in physically inactive pregnant women. Matern Child Health J. 2016 May;20(5):1091-101. PMID: 26649879. doi: 10.1007/s10995-015-1895-7.

28. Hooke MC, Gilchrist L, Tanner L, Hart N, Withycombe JS. Use of a fitness tracker to promote physical activity in children with acute lymphoblastic leukemia. Pediatr Blood Cancer. 2016 Apr;63(4):684-9. PMID: 26756736. doi: 10.1002/pbc.25860.

29. King AC, Ahn DK, Oliveira BM, Atienza AA, Castro CM, Gardner CD. Promoting physical activity through hand-held computer technology. Am J Prev Med. 2008 Feb;34(2):138-42. PMID: 18201644. doi: 10.1016/j.amepre.2007.09.025.

30. Martin SS, Feldman DI, Blumenthal RS, Jones SR, Post WS, McKibben RA, Michos ED, Ndumele CE, Ratchford EV, Coresh J, Blaha MJ. Mactive: A randomized clinical trial of an automated mHealth intervention for physical activity promotion. J Am Heart Assoc. 2015 Nov 09;4(11). PMID: 26553211. doi: 10.1161/JAHA.115.002239.

31. McManus AM, Masters RS, Laukkanen RM, Yu CC, Sit CH, Ling FC. Using heart-rate feedback to increase physical activity in children. Prev Med. 2008 Oct;47(4):402-8. PMID: 18590757. doi: 10.1016/j.ypmed.2008.06.001.

32. Patel MS, Volpp KG, Rosin R, Bellamy SL, Small DS, Fletcher MA, Osman-Koss R, Brady JL, Haff N, Lee SM, Wesby L, Hoffer K, Shuttleworth D, Taylor DH, Hilbert V, Zhu J, Yang L, Wang X, Asch DA. A randomized trial of social comparison feedback and financial incentives to increase physical activity. Am J Health Promot. 2016 Jul;30(6):416-24. PMID: 27422252. doi: 10.1177/0890117116658195.

33. Paul L, Wyke S, Brewster S, Sattar N, Gill JM, Alexander G, Rafferty D, McFadyen AK, Ramsay A, Dybus A. Increasing physical activity in stroke survivors using starfish, an interactive mobile phone application: A pilot study. Top Stroke Rehabil. 2016 Jun;23(3):170-7. PMID: 27077973. doi: 10.1080/10749357.2015.1122266.

34. Prestwich A, Conner M, Hurling R, Ayres K, Morris B. An experimental test of control theory-based interventions for physical activity. Br J Health Psychol. 2016 Nov;21(4):812-26. PMID: 27169809. doi: 10.1111/bjhp.12198.

35. van der Weegen S, Verwey R, Spreeuwenberg M, Tange H, van der Weijden T, de Witte L. It's life! Mobile and web-based monitoring and feedback tool embedded in primary care increases physical activity: A cluster randomized controlled trial. J Med Internet Res. 2015 Jul 24;17(7):e184. PMID: 26209025. doi: 10.2196/jmir.4579.

36. van Nimwegen M, Speelman AD, Overeem S, van de Warrenburg BP, Smulders K, Dontje ML, Borm GF, Backx FJ, Bloem BR, Munneke M, ParkFit Study G. Promotion of physical activity and fitness in sedentary patients with parkinson's disease: Randomised controlled trial. BMJ. 2013 Mar 01;346:f576. PMID: 23457213. doi: 10.1136/bmj.f576.

37. Walsh JC, Corbett T, Hogan M, Duggan J, McNamara A. An mhealth intervention using a smartphone app to increase walking behavior in young adults: A pilot study. JMIR mHealth and uHealth. 2016 Sep 22;4(3):e109. PMID: 27658677. doi: 10.2196/mhealth.5227.

38. Wijsman CA, Westendorp RG, Verhagen EA, Catt M, Slagboom PE, de Craen AJ, Broekhuizen K, van Mechelen W, van Heemst D, van der Ouderaa F, Mooijaart SP. Effects of a web-based intervention on physical activity and metabolism in older adults: Randomized controlled trial. J Med Internet Res. 2013 Nov 06;15(11):e233. PMID: 24195965. doi: 10.2196/jmir.2843.

39. Burke LE, Styn MA, Sereika SM, Conroy MB, Ye L, Glanz K, Sevick MA, Ewing LJ. Using mhealth technology to enhance self-monitoring for weight loss: A randomized trial. Am J Prev Med. 2012 Jul;43(1):20-6. PMID: 22704741. doi: 10.1016/j.amepre.2012.03.016.

40. Collins CE, Morgan PJ, Jones P, Fletcher K, Martin J, Aguiar EJ, Lucas A, Neve MJ, Callister R. A 12-week commercial web-based weight-loss program for overweight and obese adults: Randomized controlled trial comparing basic versus enhanced features. J Med Internet Res. 2012 Apr 25;14(2):e57. PMID: 22555246. doi: 10.2196/jmir.1980.

41. Fassnacht DB, Ali K, Silva C, Goncalves S, Machado PP. Use of text messaging services to promote health behaviors in children. J Nutr Educ Behav. 2015 Jan-Feb;47(1):75-80. PMID: 25282200. doi: 10.1016/j.jneb.2014.08.006.

42. Fujii H, Nakade M, Haruyama Y, Fukuda H, Hashimoto M, Ikuyama T, Kaburagi H, Murai E, Okumura M, Sairenchi T, Muto T. Evaluation of a computer-tailored lifestyle modification support tool for employees in Japan. Ind Health. 2009 Jul;47(3):333-41. PMID: 19531920.

43. Jakicic JM, Davis KK, Rogers RJ, King WC, Marcus MD, Helsel D, Rickman AD, Wahed AS, Belle SH. Effect of wearable technology combined with a lifestyle intervention on long-term weight loss: The IDEA randomized clinical trial. JAMA. 2016 Sep 20;316(11):1161-71. PMID: 27654602. doi: 10.1001/jama.2016.12858.

44. Khanna R, Stoddard PJ, Gonzales EN, Villagran-Flores M, Thomson J, Bayard P, Palos Lucio AG, Schillinger D, Bertozzi S, Gonzales R. An automated telephone nutrition support system for spanish-speaking patients with diabetes. Journal of diabetes science and technology. 2014 Nov;8(6):1115-20. PMID: 25239122. doi: 10.1177/1932296814550186.

45. Lim S, Kang SM, Kim KM, Moon JH, Choi SH, Hwang H, Jung HS, Park KS, Ryu JO, Jang HC. Multifactorial intervention in diabetes care using real-time monitoring and tailored feedback in type 2 diabetes. Acta Diabetol. 2016 Apr;53(2):189-98. PMID: 25936739. doi: 10.1007/s00592-015-0754-8.

46. Nollen NL, Mayo MS, Carlson SE, Rapoff MA, Goggin KJ, Ellerbeck EF. Mobile technology for obesity prevention: A randomized pilot study in racial- and ethnic-minority girls. Am J Prev Med. 2014 Apr;46(4):404-8. PMID: 24650843. doi: 10.1016/j.amepre.2013.12.011.

47. Sacks FM, Bray GA, Carey VJ, Smith SR, Ryan DH, Anton SD, McManus K, Champagne CM, Bishop LM, Laranjo N, Leboff MS, Rood JC, de Jonge L, Greenway FL, Loria CM, Obarzanek E, Williamson DA. Comparison of weight-loss diets with different compositions of fat, protein, and carbohydrates. N Engl J Med. 2009 Feb 26;360(9):859-73. PMID: 19246357. doi: 10.1056/NEJMoa0804748.

48. Shapiro JR, Bauer S, Hamer RM, Kordy H, Ward D, Bulik CM. Use of text messaging for monitoring sugar-sweetened beverages, physical activity, and screen time in children: A

pilot study. J Nutr Educ Behav. 2008 Nov-Dec;40(6):385-91. PMID: 18984496. doi: 10.1016/j.jneb.2007.09.014.

49. Shuger SL, Barry VW, Sui X, McClain A, Hand GA, Wilcox S, Meriwether RA, Hardin JW, Blair SN. Electronic feedback in a diet- and physical activity-based lifestyle intervention for weight loss: A randomized controlled trial. Int J Behav Nutr Phy. 2011 May 18;8:41. PMID: 21592351. doi: 10.1186/1479-5868-8-41.

50. Steinberg DM, Levine EL, Askew S, Foley P, Bennett GG. Daily text messaging for weight control among racial and ethnic minority women: Randomized controlled pilot study. J Med Internet Res. 2013 Nov 18;15(11):e244. PMID: 24246427. doi: 10.2196/jmir.2844.

51. Willey S, Walsh JK. Outcomes of a mobile health coaching platform: 12-week results of a single-arm longitudinal study. JMIR mHealth and uHealth. 2016 Jan 08;4(1):e3. PMID: 26747611. doi: 10.2196/mhealth.4933.
