# Supplementary material for: Just-in-Time Feedback in Diet and Physical Activity Interventions: Systematic Review and Practical Design Framework
Source: J Med Internet Res. 2018 Mar 22;20(3):e106. doi: 10.2196/jmir.8701 (PMC5887039; doi:10.2196/jmir.8701)
Supplement: Multimedia Appendix 5 [file jmir_v20i3e106_app5.pdf]

Table 1. Summary of outcomes and feedback efficacy for diet only intervention studies. Significant: *P*<.05.

| Author, year | Group | Targeted behavior and weight change outcomes | | | |
|---|---|---|---|---|---|
| Ford, 2010 [21] | | Body mass index (BMI; kg/m$^2$) | | | |
| | Mandometer training | −0.40 | | | |
| | Standard care | −0.14 | | | |
| | *Between-group difference*[a] | *Significant* | | | |
| Mummah, 2016 [22] | | Vegetable intake (servings/day) | Green leafy vegetable intake (servings/day) | Cruciferous vegetable intake (servings/day) | Dark yellow vegetable intake (servings/day) |
| | Vegethon mobile app | Adjusted mean difference=7.4 servings | Adjusted mean difference=2.6 servings | Adjusted mean difference=1.6 servings | Adjusted mean difference=0.8 servings |
| | Waitlist control | | | | |
| | *Between-group difference*[a] | *Significant* | *Significant* | *Significant* | *Significant* |
| Pederson, 2016 [23] | | Change in F/V intake behaviors | | | |
| | SMS text messages (short service message, SMS) + nutrition education | −0.07 | | | |
| | SMS text messages | −0.05 | | | |
| | Minimal intervention control | −0.18 | | | |
| | *Between-group difference*[a] | *Not significant (NS)* | | | |

[a]*P*-values for between-group differences may not have been reported in the cited study. Notation of significance or nonsignificance has been provided for consistency across studies.
[b]Primary outcome.
[c]Significant within-group differences were reported.
[d,e,f]Significant differences among three or more groups denoted by different superscripts.

Table 2. Summary of outcomes and feedback efficacy for PA sedentary behavior only intervention studies. Significant: *P*<.05.

| Author, year | Group | Targeted behavior and weight change outcomes | | | |
|---|---|---|---|---|---|
| Adams, 2013 [24] | | Average steps/day | | | |
| | Adaptive goals + performance feedback | +2728[c] | | | |
| | Static goals + encouraging | +1598[c] | | | |

| | | | | | |
|---|---|---|---|---|---|
| | messages | | | | |
| | *Between-group difference[a]* | *Significant* | | | |
| Bond, 2014 [25] | | Sedentary[a] (min/day) | Light physical activity (PA) (min/day) | Moderate-to-vigorous physical activity (MVPA; min/day) | |
| | 3-min activity break | −47.2[d] | +31.0[d] | +16.3 | |
| | 6-min activity break | −44.5[e] | +31.0[e] | +13.5 | |
| | 12-min activity break | −26.2[f] | +15.3[f] | +10.8 | |
| | *Between-group difference[a]* | *Significant* | *Significant* | *NS* | |
| Chapman, 2016 [26] | | Average steps/day | Self-reported walking (min/week) | | |
| | Step tracking + feedback | +1154 | +89 | | |
| | Step tracking only | +832 | +78 | | |
| | *Between-group difference[a]* | *NS* | *NS* | | |
| Choi, 2016 [27] | | Average steps/day (1-4 weeks) | Average steps/day (5-8 weeks) | Average steps/day (9-12 weeks) | TV or computer time (hours/week) |
| | FitBit + feedback | +1166.6 | +1092.1 | +1096.2 | −10.8 |
| | FitBit only | +1062.6 | +804.3 | +258.7 | −6.3 |
| | *Between-group difference[a]* | *NS* | *NS* | *NS* | *NS* |
| Hooke, 2016 [28] | | Average steps/day Baseline to week-2 | Average steps/day week-2 to week-1 | Average steps/day week-1 to steroid pulse | |
| | FitBit + emails | −23 | +269 | −307 | |
| | *Between-group difference[a]* | *Not applicable (N/A)* | *N/A* | *N/A* | |
| King, 2008 [29] | | Moderate-intensity activity (min/week) | Caloric expenditure (kcal/kg/week) | Caloric expenditure (kcal/week) | |
| | Personal digital assistant (PDA) intervention | 310.6 | 19.1 | 1653.9 | |
| | Waitlist control | 125.5 | 7.8 | 1406.8 | |
| | *Between-group difference[a]* | *Significant* | *NS* | *Significant* | |
| Martin, 2015 [30] | | Phase 2: average steps/day[b] | Phase 2: activity time (min/day) | Phase 2: aerobic time (min/day) | |
| | Unblinded with | +2334[d] | +21[d] | −13[d] | |

| | | | | | |
|---|---|---|---|---|---|
| | phase 2 texts | | | | |
| | Unblinded without phase 2 texts | −200[f] | 0[f] | −1[f] | |
| | Blinded to activity tracking | −1042[f] | −8[f] | −3[f] | |
| | *Between-group difference[a]* | *Significant* | *Significant* | *Significant* | |
| McManus, 2008 [31] | | Time above heart rate (HR) target (min/day) | Time moderately active (min/day) | Time vigorously active (min/day) | |
| | Education + feedback school | +86[c] | +4.6 | +5.4[c] | |
| | No education + feedback school | +69[c] | +0.5 | +7.1[c] | |
| | Control school | *Not reported (NR)* | *NR* | *NR* | |
| | *Between-group difference[a]* | *NS* | *NS* | *NS* | |
| Patel, 2016 [32] | | Mean proportion meeting 7000-step/day goal | | | |
| | Comparison with top quartile + incentive | 0.38[e] | | | |
| | Comparison with mean performance + incentive | 0.45[f] | | | |
| | Comparison with top quartile only | 0.27[d] | | | |
| | Comparison with mean performance only | *Significant* | | | |
| | *Between-group difference[a]* | | | | |

| | | | | | | |
|---|---|---|---|---|---|---|
| Paul, 2016 [33] | | Average steps/day[b] | Sedentary time (h)[b] | Upright time (h)[b] | Walking time (h)[b] | BMI ($kg/m^2$) |
| | STARFISH app | +1,633 | −0.9 | +1.2 | +0.3 | +0.0 |
| | Usual care control | −747 | −0.6 | +0.5 | −0.2 | +0.1 |
| | *Between-group difference[a]* | *Significant* | *NS* | *NS* | *Significant* | *NS* |

| | | | | | |
|---|---|---|---|---|---|
| Prestwich, 2016 [34] | | Energy expenditure Metabolic equivalents (MET)-min/week) | | | |
| | Goal setting + self-monitoring + feedback | 14,156[d] | | | |

| | | | | | |
|---|---|---|---|---|---|
| | Goal-setting + self-monitoring | 13,891[e] | | | |
| | Goal setting only | 13,661[f] | | | |
| | *Between-group difference*[a] | *Significant* | | | |
| van der Weegen, 2015 [35] | | MVPA (min/day) | | | |
| | Self-management support + activity monitor | +9.53[d] | | | |
| | Self-management support | −2.13[f] | | | |
| | Standard care | −1.73[f] | | | |
| | *Between-group difference*[a] | *Significant* | | | |
| van Nimweegen, 2013 [36] | | Activity by recall (median hours/week)[b] | Activity by diary (median hours/week) | Energy expenditure (median kcal/day) | |
| | ParkFit program | −0.3 | +1.3 | +38.7 | |
| | General physiology program | −1.8 | +0.5 | −14.2 | |
| | *Between-group difference*[a] | *NS* | *Significant* | *Significant* | |
| Walsh, 2016 [37] | | Step count (steps/day) | | | |
| | Pedometer + feedback | +2393[c] | | | |
| | Pedometer only | +1101[c] | | | |
| | *Between-group difference*[a] | *Significant* | | | |
| Wijsman, 2013 [38] | | Daily PA (ankle; %)[b] | Daily PA (wrist; %)[b] | MVPA (min/day)[b] | Weight (kg) |
| | DirectLife program | +46[c] | +11[c] | +11 | −1.49 |
| | Waitlist control | +12[c] | +5[c] | +0 | −0.82 |
| | *Between-group difference*[a] | *Significant* | *NS* | *Significant* | *Significant* |

[a]*P*-values for between-group differences may not have been reported in the cited study. Notation of significance or nonsignificance has been provided for consistency across studies.
[b]Primary outcome.
[c]Significant within-group differences were reported.
[d,e,f]Significant differences among three or more groups denoted by different superscripts.

Table 2. Summary of outcomes and feedback efficacy for diet and PA or sedentary behavior intervention studies. Significant: $P<.05$.

| | | | | | |
|---|---|---|---|---|---|
| Burke, 2012 [39] | | Weight (%)[b] | | | |

| | | | | | | |
|---|---|---|---|---|---|---|
| | PDA + feedback | −2.32[c] | | | | |
| | PDA only | −1.38 | | | | |
| | Paper diary | −1.94 | | | | |
| | *Between-group difference*[a] | *NS* | | | | |
| Collins, 2012 [40] | | BMI[b] (kg/$m^2$) | PA (MET min/week) | Average steps/day | Energy intake (kJ/day) | |
| | Enhanced program | −0.98[c,d] | +491.6 | +867[d] | −1465[f] | |
| | Basic program | −0.72[c,d] | +151.4 | +153[d] | −1,003[d] | |
| | Waitlist control | +0.15[f] | +341.8 | −61[f] | −734[d] | |
| | *Between-group difference*[a] | *Significant* | *NS* | *Significant* | *Significant* | |
| Fassnacht, 2015 [41] | | Adherence to monitoring[b] (% of days) | F/V intake (portions/day) | PA (hours/day) | Screen time (hours/day) | |
| | Monitoring + feedback | 61 | +0.8 | −0.1 | −0.3 | |
| | Education sessions | N/A | −0.3 | −0.5 | −0.5 | |
| | *Between-group difference*[a] | *N/A* | *Significant* | *NS* | *Significant* | |
| Fujii, 2009 [42] | | Low salt intake (% score≥6) | Low energy intake (% score≥2) | F/V intake (% score≥1) | Healthy eating (% score≥3) | Regular exercise (% ≥2 d/week) |
| | Health Up Navi, Men | +9.1 | +12.7[c] | +8.3 | +4.2 | +12.4[c] |
| | Health Up Navi, Women | +11.7[c] | +1.4 | +7.9 | −4.8 | +14.1[c] |
| | *Between-group difference*[a] | *N/A* | *N/A* | *N/A* | *N/A* | *N/A* |
| Jakicic, 2016 [43] | | Weight (kg)[b] | Weight (%) | BMI (kg/$m^2$) | | |
| | Enhanced program + feedback | −3.5 | −3.6 | −1.1 | | |
| | Standard program | −5.9 | −6.4 | −1.8 | | |
| | *Between-group difference*[a] | *Significant* | *Significant* | *NS* | | |
| Khanna, 2014 [44] | | Glycated hemoglobin (HbA1c), %[b] | BMI (kg/$m^2$) | | | |
| | Automated nutrition support | −0.1 | +0.4 | | | |
| | Waitlist control | −0.3 | −0.1 | | | |

| | | | | | |
|---|---|---|---|---|---|
| | *Between-group difference*[a] | *NS* | *NS* | | |
| Lim, 2016 [45] | | HbA1c (% < 7.0%)[b] | BMI (kg/$m^2$) | Caloric intake (%) | Exercise frequency (episodes/week) |
| | u-healthcare | 26[c] | −0.6[c] | −8.5[c] | +2.3[c] |
| | Blood glucose self-monitoring | 12 | −0.3 | −2.4[c] | +0.2 |
| | *Between-group difference*[a] | *Significant* | *NS* | *Significant* | *Significant* |
| Nollen, 2014 [46] | | BMI Weeks 0-12 (kg/$m^2$) | F/V intake Weeks 0-4 (servings/day) | SSB intake Weeks 0-8 (servings/day) | Screen time Weeks 0-12 (hours/day) |
| | Mobile technology | −0.21 | +0.88 | −0.33 | +0.32 |
| | Educational materials only | −0.27 | −0.12 | +0.02 | +0.12 |
| | *Between-group difference*[a] | *NS* | *NS* | *NS* | *NS* |
| Sacks, 2009 [47] | | Weight (kg) | | | |
| | Diet 1: low fat, average protein | −3.0 | | | |
| | Diet 2: low fat, high protein | −3.6 | | | |
| | Diet 3: high fat, average protein | −3.3 | | | |
| | Diet 4: high fat, high protein | −3.3 | | | |
| | *Between-group difference*[a] | *NS* | | | |
| Shapiro, 2008 [48] | | Self-reported exercise (min/day) | Self-reported SSB (servings/day) | Self-reported screen time (min/day) | |
| | SMS with feedback | +34.4 | −0.9 | −68.7 | |
| | Paper diaries | +30.7 | −1.4 | −97.6 | |
| | No-monitoring control | −15.1 | −0.9 | −76.8 | |
| | *Between-group difference*[a] | *NS* | *NS* | *NS* | |
| Shuger, 2011 [49] | | Weight (kg) | | | |
| | Armband + group weight loss | −6.59[c,d] | | | |
| | Armband alone | −3.55[c,e] | | | |

| | | | | | |
|---|---|---|---|---|---|
| | Group weight loss | −1.86[c,e] | | | |
| | Standard care | −0.89[f] | | | |
| | *Between-group difference*[a] | *Significant* | | | |
| Steinberg, 2013 [50] | | Weight (kg) | Weight (%) | BMI (kg/m$^2$) | |
| | Shape Plan | −1.27 | −0.97 | −0.47 | |
| | Educational materials | +1.14 | +1.32 | +0.42 | |
| | *Between-group difference*[a] | *NS* | *NS* | *NS* | |
| Willey, 2016 [51] | | Weight (lb) | Weight (%) | | |
| | YouPlus Health app | −13.5[c] | −7.3%[c] | | |
| | *Between-group difference*[a] | *N/A* | *N/A* | | |

[a]*P*-values for between-group differences may not have been reported in the cited study. Notation of significance or nonsignificance has been provided for consistency across studies.
[b]Primary outcome.
[c]Significant within-group differences were reported.
[d,e,f]Significant differences among three or more groups denoted by different superscripts.

**References**


21. Ford AL, Bergh C, Sodersten P, Sabin MA, Hollinghurst S, Hunt LP, Shield JP. Treatment of childhood obesity by retraining eating behaviour: Randomised controlled trial. BMJ. 2009 Jan 05;340:b5388. PMID: 20051465. doi: 10.1136/bmj.b5388.
22. Mummah SA, Mathur M, King AC, Gardner CD, Sutton S. Mobile technology for vegetable consumption: A randomized controlled pilot study in overweight adults. JMIR mHealth and uHealth. 2016 May 18;4(2):e51. PMID: 27193036. doi: 10.2196/mhealth.5146.
23. Pedersen S, Gronhoj A, Thogersen J. Texting your way to healthier eating? Effects of participating in a feedback intervention using text messaging on adolescents' fruit and vegetable intake. Health Educ Res. 2016 Apr;31(2):171-84. PMID: 26850061. doi: 10.1093/her/cyv104.
24. Adams MA, Sallis JF, Norman GJ, Hovell MF, Hekler EB, Perata E. An adaptive physical activity intervention for overweight adults: A randomized controlled trial. PLoS One. 2013;8(12):e82901. PMID: 24349392. doi: 10.1371/journal.pone.0082901.
25. Bond DS, Thomas JG, Raynor HA, Moon J, Sieling J, Trautvetter J, Leblond T, Wing RR. B-mobile--a smartphone-based intervention to reduce sedentary time in overweight/obese individuals: A within-subjects experimental trial. PLoS One. 2014;9(6):e100821. PMID: 24964010. doi: 10.1371/journal.pone.0100821.
26. Chapman GB, Colby H, Convery K, Coups EJ. Goals and social comparisons promote walking behavior. Med Decis Making. 2016 May;36(4):472-8. PMID: 26139447. doi: 10.1177/0272989X15592156.
27. Choi J, Lee JH, Vittinghoff E, Fukuoka Y. mHealth physical activity intervention: A randomized pilot study in physically inactive pregnant women. Matern Child Health J. 2016 May;20(5):1091-101. PMID: 26649879. doi: 10.1007/s10995-015-1895-7.

28. Hooke MC, Gilchrist L, Tanner L, Hart N, Withycombe JS. Use of a fitness tracker to promote physical activity in children with acute lymphoblastic leukemia. Pediatr Blood Cancer. 2016 Apr;63(4):684-9. PMID: 26756736. doi: 10.1002/pbc.25860.
29. King AC, Ahn DK, Oliveira BM, Atienza AA, Castro CM, Gardner CD. Promoting physical activity through hand-held computer technology. Am J Prev Med. 2008 Feb;34(2):138-42. PMID: 18201644. doi: 10.1016/j.amepre.2007.09.025.
30. Martin SS, Feldman DI, Blumenthal RS, Jones SR, Post WS, McKibben RA, Michos ED, Ndumele CE, Ratchford EV, Coresh J, Blaha MJ. Mactive: A randomized clinical trial of an automated mHealth intervention for physical activity promotion. J Am Heart Assoc. 2015 Nov 09;4(11). PMID: 26553211. doi: 10.1161/JAHA.115.002239.
31. McManus AM, Masters RS, Laukkanen RM, Yu CC, Sit CH, Ling FC. Using heart-rate feedback to increase physical activity in children. Prev Med. 2008 Oct;47(4):402-8. PMID: 18590757. doi: 10.1016/j.ypmed.2008.06.001.
32. Patel MS, Volpp KG, Rosin R, Bellamy SL, Small DS, Fletcher MA, Osman-Koss R, Brady JL, Haff N, Lee SM, Wesby L, Hoffer K, Shuttleworth D, Taylor DH, Hilbert V, Zhu J, Yang L, Wang X, Asch DA. A randomized trial of social comparison feedback and financial incentives to increase physical activity. Am J Health Promot. 2016 Jul;30(6):416-24. PMID: 27422252. doi: 10.1177/0890117116658195.
33. Paul L, Wyke S, Brewster S, Sattar N, Gill JM, Alexander G, Rafferty D, McFadyen AK, Ramsay A, Dybus A. Increasing physical activity in stroke survivors using starfish, an interactive mobile phone application: A pilot study. Top Stroke Rehabil. 2016 Jun;23(3):170-7. PMID: 27077973. doi: 10.1080/10749357.2015.1122266.
34. Prestwich A, Conner M, Hurling R, Ayres K, Morris B. An experimental test of control theory-based interventions for physical activity. Br J Health Psychol. 2016 Nov;21(4):812-26. PMID: 27169809. doi: 10.1111/bjhp.12198.

35. van der Weegen S, Verwey R, Spreeuwenberg M, Tange H, van der Weijden T, de Witte L. It's life! Mobile and web-based monitoring and feedback tool embedded in primary care increases physical activity: A cluster randomized controlled trial. J Med Internet Res. 2015 Jul 24;17(7):e184. PMID: 26209025. doi: 10.2196/jmir.4579.
36. van Nimwegen M, Speelman AD, Overeem S, van de Warrenburg BP, Smulders K, Dontje ML, Borm GF, Backx FJ, Bloem BR, Munneke M, ParkFit Study G. Promotion of physical activity and fitness in sedentary patients with parkinson's disease: Randomised controlled trial. BMJ. 2013 Mar 01;346:f576. PMID: 23457213. doi: 10.1136/bmj.f576.
37. Walsh JC, Corbett T, Hogan M, Duggan J, McNamara A. An mhealth intervention using a smartphone app to increase walking behavior in young adults: A pilot study. JMIR mHealth and uHealth. 2016 Sep 22;4(3):e109. PMID: 27658677. doi: 10.2196/mhealth.5227.
38. Wijsman CA, Westendorp RG, Verhagen EA, Catt M, Slagboom PE, de Craen AJ, Broekhuizen K, van Mechelen W, van Heemst D, van der Ouderaa F, Mooijaart SP. Effects of a web-based intervention on physical activity and metabolism in older adults: Randomized controlled trial. J Med Internet Res. 2013 Nov 06;15(11):e233. PMID: 24195965. doi: 10.2196/jmir.2843.
39. Burke LE, Styn MA, Sereika SM, Conroy MB, Ye L, Glanz K, Sevick MA, Ewing LJ. Using mhealth technology to enhance self-monitoring for weight loss: A randomized trial. Am J Prev Med. 2012 Jul;43(1):20-6. PMID: 22704741. doi: 10.1016/j.amepre.2012.03.016.
40. Collins CE, Morgan PJ, Jones P, Fletcher K, Martin J, Aguiar EJ, Lucas A, Neve MJ, Callister R. A 12-week commercial web-based weight-loss program for overweight and obese adults: Randomized controlled trial comparing basic versus enhanced features. J Med Internet Res. 2012 Apr 25;14(2):e57. PMID: 22555246. doi: 10.2196/jmir.1980.
41. Fassnacht DB, Ali K, Silva C, Goncalves S, Machado PP. Use of text messaging services to promote health behaviors in children. J Nutr Educ Behav. 2015 Jan-Feb;47(1):75-80. PMID: 25282200. doi: 10.1016/j.jneb.2014.08.006.

42. Fujii H, Nakade M, Haruyama Y, Fukuda H, Hashimoto M, Ikuyama T, Kaburagi H, Murai E, Okumura M, Sairenchi T, Muto T. Evaluation of a computer-tailored lifestyle modification support tool for employees in Japan. Ind Health. 2009 Jul;47(3):333-41. PMID: 19531920.
43. Jakicic JM, Davis KK, Rogers RJ, King WC, Marcus MD, Helsel D, Rickman AD, Wahed AS, Belle SH. Effect of wearable technology combined with a lifestyle intervention on long-term weight loss: The IDEA randomized clinical trial. JAMA. 2016 Sep 20;316(11):1161-71. PMID: 27654602. doi: 10.1001/jama.2016.12858.
44. Khanna R, Stoddard PJ, Gonzales EN, Villagran-Flores M, Thomson J, Bayard P, Palos Lucio AG, Schillinger D, Bertozzi S, Gonzales R. An automated telephone nutrition support system for spanish-speaking patients with diabetes. Journal of diabetes science and technology. 2014 Nov;8(6):1115-20. PMID: 25239122. doi: 10.1177/1932296814550186.
45. Lim S, Kang SM, Kim KM, Moon JH, Choi SH, Hwang H, Jung HS, Park KS, Ryu JO, Jang HC. Multifactorial intervention in diabetes care using real-time monitoring and tailored feedback in type 2 diabetes. Acta Diabetol. 2016 Apr;53(2):189-98. PMID: 25936739. doi: 10.1007/s00592-015-0754-8.
46. Nollen NL, Mayo MS, Carlson SE, Rapoff MA, Goggin KJ, Ellerbeck EF. Mobile technology for obesity prevention: A randomized pilot study in racial- and ethnic-minority girls. Am J Prev Med. 2014 Apr;46(4):404-8. PMID: 24650843. doi: 10.1016/j.amepre.2013.12.011.
47. Sacks FM, Bray GA, Carey VJ, Smith SR, Ryan DH, Anton SD, McManus K, Champagne CM, Bishop LM, Laranjo N, Leboff MS, Rood JC, de Jonge L, Greenway FL, Loria CM, Obarzanek E, Williamson DA. Comparison of weight-loss diets with different compositions of fat, protein, and carbohydrates. N Engl J Med. 2009 Feb 26;360(9):859-73. PMID: 19246357. doi: 10.1056/NEJMoa0804748.
48. Shapiro JR, Bauer S, Hamer RM, Kordy H, Ward D, Bulik CM. Use of text messaging for monitoring sugar-sweetened beverages, physical activity, and screen time in children: A

pilot study. J Nutr Educ Behav. 2008 Nov-Dec;40(6):385-91. PMID: 18984496. doi: 10.1016/j.jneb.2007.09.014.

49. Shuger SL, Barry VW, Sui X, McClain A, Hand GA, Wilcox S, Meriwether RA, Hardin JW, Blair SN. Electronic feedback in a diet- and physical activity-based lifestyle intervention for weight loss: A randomized controlled trial. Int J Behav Nutr Phy. 2011 May 18;8:41. PMID: 21592351. doi: 10.1186/1479-5868-8-41.

50. Steinberg DM, Levine EL, Askew S, Foley P, Bennett GG. Daily text messaging for weight control among racial and ethnic minority women: Randomized controlled pilot study. J Med Internet Res. 2013 Nov 18;15(11):e244. PMID: 24246427. doi: 10.2196/jmir.2844.

51. Willey S, Walsh JK. Outcomes of a mobile health coaching platform: 12-week results of a single-arm longitudinal study. JMIR mHealth and uHealth. 2016 Jan 08;4(1):e3. PMID: 26747611. doi: 10.2196/mhealth.4933.
